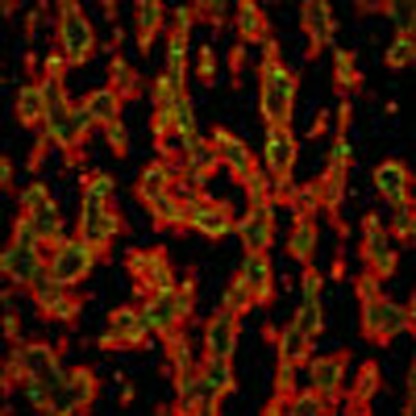# Identification of platelet function defects by multi-parameter assessment of thrombus formation

**DOI:** 10.1038/ncomms5257

**Published:** 2014-07-16

**Authors:** Susanne M. de Witt, Frauke Swieringa, Rachel Cavill, Moniek M. E. Lamers, Roger van Kruchten, Tom Mastenbroek, Constance Baaten, Susan Coort, Nicholas Pugh, Ansgar Schulz, Inge Scharrer, Kerstin Jurk, Barbara Zieger, Kenneth J. Clemetson, Richard W. Farndale, Johan W. M. Heemskerk, Judith M.E.M. Cosemans

**Affiliations:** 1 Department of Biochemistry, Cardiovascular Research Institute Maastricht (CARIM), Maastricht University, Universiteitssingel 50, 6229 ER Maastricht, The Netherlands; 2 Department of Toxicogenomics, Maastricht University, Universiteitssingel 50, 6229 ER Maastricht, The Netherlands; 3 Department of Bioinformatics, Maastricht University, Universiteitssingel 50, 6229 ER Maastricht, The Netherlands; 4 Anglia Ruskin University, East Rd, Cambridge CB11PT, UK; 5 Pediatric Centre, University Clinic Ulm, Universitätsklinik Für Kinder- und Jugendmedizin, Eythstrasse 24, D-89075 Ulm, Germany; 6 Center for Thrombosis and Haemostasis (CTH), Universitätsmedizin der Johannes Gutenberg, University of Mainz, Langenbeckstrasse 1, 55131 Mainz, Germany; 7 Department of Pediatrics and Adolescent Medicine, University Medical Centre Freiburg, Mathildenstrasse 1, 79106 Freiburg, Germany; 8 Department of Haematology, Inselspital, University of Bern, CH-3010 Bern, Switzerland; 9 Department of Biochemistry, University of Cambridge, Downing Site, Cambridge CB2 1QW, UK; 10 These authors contributed equally to this work

## Abstract

Assays measuring platelet aggregation (thrombus formation) at arterial shear rate mostly use collagen as only platelet-adhesive surface. Here we report a multi-surface and multi-parameter flow assay to characterize thrombus formation in whole blood from healthy subjects and patients with platelet function deficiencies. A systematic comparison is made of 52 adhesive surfaces with components activating the main platelet-adhesive receptors, and of eight output parameters reflecting distinct stages of thrombus formation. Three types of thrombus formation can be identified with a predicted hierarchy of the following receptors: glycoprotein (GP)VI, C-type lectin-like receptor-2 (CLEC-2)>GPIb>α_6_β_1_, α_IIb_β_3_>α_2_β_1_>CD36, α_5_β_1_, α_v_β_3_. Application with patient blood reveals distinct abnormalities in thrombus formation in patients with severe combined immune deficiency, Glanzmann’s thrombasthenia, Hermansky–Pudlak syndrome, May–Hegglin anomaly or grey platelet syndrome. We suggest this test may be useful for the diagnosis of patients with suspected bleeding disorders or a pro-thrombotic tendency.

## Abstract (web summary)

Platelets from patients with bleeding disorders often display altered adherence to surface proteins. In this study, de Witt *et al.* design a flow chamber for the systematic interrogation of platelets attaching to 52 adhesive surfaces, which may be helpful for the diagnosis of platelet disorders.

The fundamental role of platelets in haemostasis and thrombosis relies on their capability of adhesion to specific locations of the perturbed vessel wall upon injury, damage or inflammation. Continued adhesion of flowing platelets leads to buildup of a platelet plug or thrombus and is required to stop bleeding or, under pathological conditions, to induce thrombosis, for instance after rupture of an atherosclerotic plaque^1^. Many experimental studies with genetically modified mice or with blood from patients with haemostatic deficiencies, performed at either arterial (high shear rate) or venous (low shear rate) flow conditions, have emphasized that thrombus formation is a complex process encompassing multiple platelet receptors and signalling mechanisms^2^,^3^,^4^.

For over two decades, parallel-plate flow chambers have been used to measure platelet adhesion and activation under arterial or venous flow conditions, in particular using surfaces such as extracellular matrix or collagen^5^,^6^. Currently, this process of platelet adhesion and aggregation in flow devices is described as flow-dependent thrombus formation, regardless of the presence or absence of anticoagulants^4^. Whole-blood flow chamber tests with blood from many strains of genetically modified mice have revealed platelet function defects under flow *in vitro* that often associate with a reduced arterial thrombosis tendency *in vivo*^1^,^7^,^8^. These outcomes have boosted the use of commercial and home-made flow devices, in particular for the assessment of human platelet activity in preclinical settings, in spite of the fact that international recommendations stress the need for further standardization of devices, protocols and measurement parameters^9^. In recent years, various types of microfluidic devices have been developed requiring only small volumes of human blood^10^,^11^, including devices containing endothelium, for instance to study blood from patients with sickle cell disease^12^. Unfortunately, however, the great variation in design and use of the microfluidic chips hinders the process of standardization^13^. On the other hand, relatively simple, one-parameter microfluidics tests using collagen surfaces have already been employed to determine inter-subject variability and the efficacy of antiplatelet therapy in cardiac patients^14^,^15^,^16^,^17^.

Clinically, the PFA-100 is the only device currently validated that assesses platelet function under high-shear flow conditions, by measuring the occlusion time due to platelet aggregation on a collagen matrix. The PFA-100 is frequently utilized to evaluate deficiencies in platelet function or von Willebrand factor (vWF) activity, but it only provides a single end-stage parameter. Current guidelines for laboratory investigations to check for heritable disorders of platelet function recommend the PFA-100 as an optional screening test, but also stipulate that this test is not diagnostic and is insensitive to mild platelet disorders^18^. Taken together, there are promising possibilities for clinical employment of flow assays to test platelet adhesion and aggregation, but current methods often are incompletely developed and insufficiently standardized.

The classic concept of flow-dependent thrombus formation is based on collagen-dependent models of platelet aggregation, both *in vivo* in damaged mouse vessels and *in vitro* using collagen-coated flow devices. Fibrillar collagen (collagen I or III) is considered as the primary platelet-activating substance in the damaged vessel wall controlling the thrombotic process^2^,^4^,^8^,^19^,^20^. The concept, in brief, is that at high arterial wall shear rate, initial platelet rolling is regulated by the interaction of platelet glycoprotein Ib-V-IX (GPIb) to vWF which is bound to collagen. Platelet adhesion and activation by vWF/collagen then is enforced by interplay of the collagen receptors, glycoprotein VI (GPVI) and integrin α_2_β_1_, and the fibrinogen receptor, integrin α_IIb_β_3_ (refs 21, 22). Platelets may first adhere via integrins and then become activated via GPVI, or first interact with GPVI^23^. Collagen-induced activation of platelets includes rises in cytosolic Ca^2+^, secretion of dense and α-granules (monitored as P-selectin expression) and release of autacoids such as ADP, ATP and thromboxane A_2_, thus resulting in a plethora of paracrine substances able of recruiting and capturing other passing platelets. Together with GPVI, these mediators induce affinity changes in integrins α_2_β_1_ and α_IIb_β_3_, which are required for thrombus stability^24^,^25^. Additionally, GPVI signalling stimulates platelet procoagulant activity and thrombin generation via phosphatidylserine exposure^26^.

However, in the perturbed vessel wall, platelets will be in contact with many other adhesive ligands than only collagen and vWF. Platelets indeed express adhesive receptors for a large number of vascular and plasma proteins^2^. So far, adhesion of platelets under flow conditions has been studied on surfaces coated with fibrinogen^27^, fibronectin^28^, vitronectin^29^,^30^, osteopontin, laminin^31^,^32^ and thrombospondin-1 (ref. 33). Another relevant receptor is the C-type lectin-like receptor (CLEC-2), which still lacks a clear physiological ligand, but yet supports in murine studies thrombus formation in a similar way to the collagen receptor, GPVI^34^,^35^,^36^. Precisely how these adhesive proteins and receptors support thrombus formation under flow conditions in comparison with collagen and GPVI remains poorly understood.

In this paper, we present a first systematic study to compare key physiological platelet-adhesive proteins for all major adhesive receptors, alone and in combination, to support whole-blood thrombus formation at specified wall shear rates. Based on this inventory, we developed a multi-microspot test using nine different surfaces, which we validated using blood samples from patients with distinct platelet function deficiencies. Using systems biology approaches, we employed the test outcomes for a model predicting the roles of various receptors in thrombus formation at high and low wall shear rate, and for a template determining aberrations in this process in patients with platelet dysfunctions.

## Results

### Arrays of adhesive surfaces to assess thrombus formation

**Figure 1:**
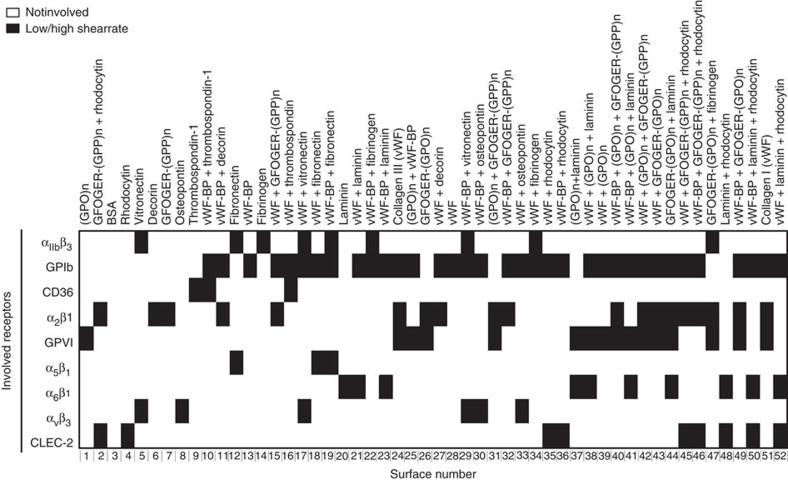
Protein surfaces used for flow studies and assignment map of platelet receptors interacting with indicated proteins. Assignment of interactions of platelet receptors to immobilized protein or peptide ligands was as represented in Supplementary Table 1. Surfaces were numbered 1–52, based on unsupervised hierarchical cluster analysis of thrombus parameters (see Fig. 4). Colour code: white, not involved; black, involved at low/high wall shear rate.

To compare the roles of established platelet-adhesive receptors in thrombus formation, we selected ligands of these receptors that are present in either the vessel wall or platelet aggregates. In addition, we used a number of chemically synthesized peptides binding to the same receptors (Supplementary Table 1). In preliminary experiments, we used different coating concentrations to assure that the selected proteins and peptides: (i) remained bound to degreased glass coverslips after repeated rinsing (Coomassie staining), and (ii) showed optimal platelet adhesion under static conditions (S.M.d.W., personal communication). Optimized surfaces for platelet adhesion consisted of the following proteins: collagens I and III, decorin, fibrinogen, fibronectin, laminin 511/521, osteopontin, thrombospondin-1, vitronectin and/or vWF (Fig. 1). Also included were the snake venom rhodocytin, four collagen-mimetic triple-helical peptides, that is, GFOGER-(GPP)_n_, GFOGER-(GPO)_n_, (GPO)_n_ and a vWF-binding peptide (vWF-BP). Immobilized bovine serum albumin (BSA) served as negative control surface. Optimally effective coating concentrations of the collagen-mimetic peptides were established before^24^,^37^.

**Figure 2:**
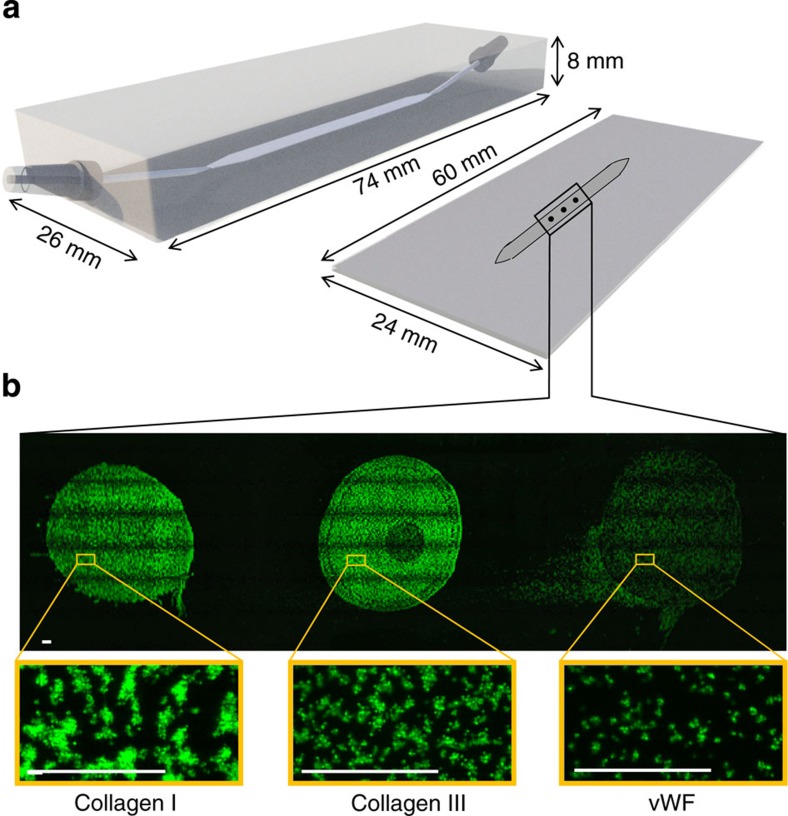
Measurement of thrombus formation on microspot arrays of platelet-adhesive surfaces. (**a**) Schematic drawing of the used parallel-plate flow chamber (3 mm width, 50 μm depth) and microspot-coated coverslip. Note the small-angular (11°) chamber inlet and outlet, preventing flow perturbations. (**b**) Distribution of DiOC_6_-labelled platelets adhered to consecutive microspots of collagen type I, collagen type III and vWF, after 3.5-min flow of blood at wall shear rate of 1,600 s^−1^. Given are tile scans of fluorescence images of the full microspots (bar, 100 μm). Black rims are image artifacts due to the tile scanning. Lower panels are enlarged images.

By coating these proteins/peptides alone or in combinations as a row of three microspots (diameter 1,000 μm, separation 2,000 μm), and mounting the coated coverslips in a standard parallel-plate perfusion chamber^38^, it was possible to determine the thrombus-forming activity of multiple surfaces at the same time (Fig. 2a). Tile scans performed after perfusion of 3,3′dihexyloxacarbocyanine iodide (DiOC_6_)-labelled whole blood indicated homogeneous adhesion of platelets to the microspots, either as single cells or in aggregates depending on the surface coating (Fig. 2b). Control experiments further established that the order of coated proteins in microspots did not affect platelet deposition. For instance, microspotting of fibrinogen, fibronectin or collagen I, at upstream or downstream positions, resulted in the same amount of platelet adhesion and aggregation (Supplementary Fig. 1). This pointed to the absence of significant paracrine cross-talk from activated platelets on adjacent microspots.

**Figure 3:**
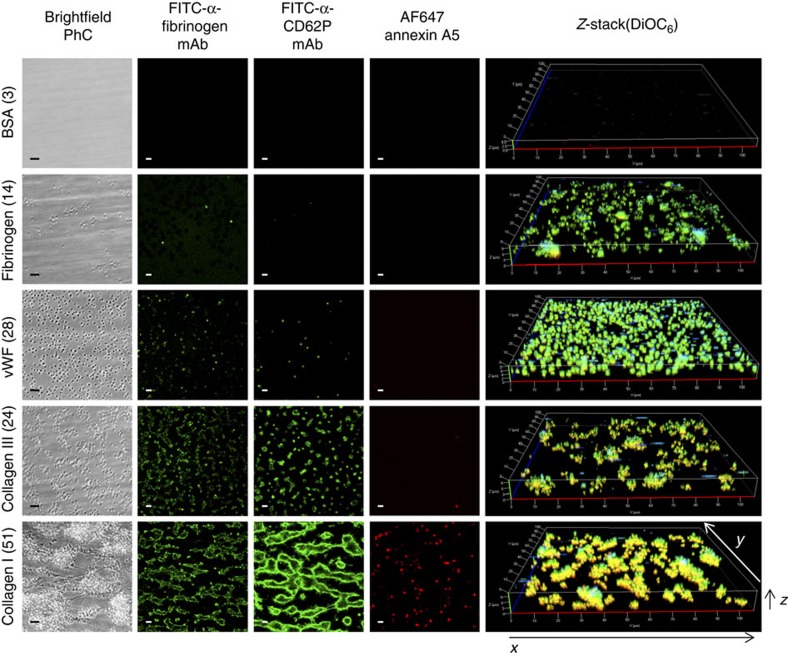
Different types of thrombi formed on various microspots. Blood from control subjects was perfused over microspots with indicated coating for 3.5 min at 1,600 s^−1^. Shown are representative microscopic images (>5 donors), from left to right: phase-contrast images of adhered platelets (i); fluorescence images of platelets binding FITC-labelled anti-fibrinogen mAb (ii), FITC-labelled anti-CD62P mAb (iii) or AF647-annexin A5 (iv); further, *z*-stacks from aggregates of DiOC_6_-labelled platelets (v). Bar, 10 μm. Images from typical surfaces are given (for all 52 surfaces, see Supplementary Fig. 1).

For a comparative analysis, whole blood from healthy subjects was perfused over microspots (three per run) containing the 52 combinations of substrate proteins and peptides at a high (arterial) wall shear rate of 1,600 s^−1^. Thrombus formation on all surfaces was measured using standardized microscopic procedures (see Methods). Stable platelet adhesion of DiOC_6_-labelled platelets was evaluated from sequential fluorescence images captured in real time during blood flow. Thrombus volume was assessed from *z*-stacks of confocal images of DiOC_6_ fluorescence at end stage. End stage, phase-contrast images were captured to determine overall platelet deposition (surface area coverage) and platelet aggregate size. Activation of the platelets per surface was resolved from confocal fluorescence images after labelling with fluorescein isothiocyanate (FITC)-labelled anti-fibrinogen monoclonal antibody (mAb) (fibrinogen binding, integrin α_IIb_β_3_ activation), FITC-anti-CD62P mAb (P-selectin expression) or AF647-annexin A5 (procoagulant activity). As illustrated in Fig. 3, platelet adhesion, aggregation and activation markedly differed between the various microspots. No platelets adhered to spots coated with BSA (negative control), while single platelets with low activation state adhered to coated fibrinogen. Small aggregates of platelets with activated integrin α_IIb_β_3_ and surface-expressed P-selectin formed on coated vWF or collagen III. Large platelet aggregates staining for all three activation markers formed on collagen I microspots, as expected^39^.

Control experiments were performed with blocking antibodies (all at 20 μg ml^−1^) to verify the involvement of specific receptors (Supplementary Table 1) to the flow-dependent deposition of platelets on microspots with single or double coatings (S.M.d.W., personal communication). These antibody experiments affirmed the previously established roles of: GPVI and α_2_β_1_ in thrombus formation on collagen I or III (10B12 Ab against GPVI^22^,^24^ and mAb 15D7 against integrin α_2_β_1_ (ref. 24)); and GPIb-V-IX for surfaces with vWF or vWF-BP (Fab 6B4 against GPIbα^24^). The blocking mAb 15D7 had a moderate reducing effect on platelet deposition on surfaces with expected contribution of α_2_β_1_ (vWF/GFOGER-(GPP)_n_, vWF/GFOGER-(GPO)_n_), that is, −42 and −28%, respectively (*n*=3, *P*<0.05). Blocking experiments with the anti-CD36 antibody Fab-152 gave a reduced platelet response to thrombospondin-1 (*n*=3, *P*<0.05), but unchanged platelet deposition to vWF/thrombospondin-1. Other blocking experiments were performed using mAb-1976 against integrin α_v_β_3_ and mAb-1969 against integrin α_5_β_1_ (20 μg ml^−1^). Single α_v_β_3_-binding (vitronectin, osteopontin) and α_5_β_1_-binding (fibronectin) surfaces did not show platelet adhesion at high shear rate, either in the presence or absence of the corresponding blocking antibody. In the presence of vWF, the antibodies caused a small but insignificant inhibition of platelet adhesion (*n*=3, *P*>0.06). Based on published analyses^22^,^24^,^37^, an assignment matrix was constructed of the contribution of individual adhesive receptors to platelet interaction with the different surfaces (Fig. 1). Herein, the well-known vWF-capturing ability of collagens I and III was taken into account.

### Identification of thrombus type by multi-parameter analysis

**Figure 4:**
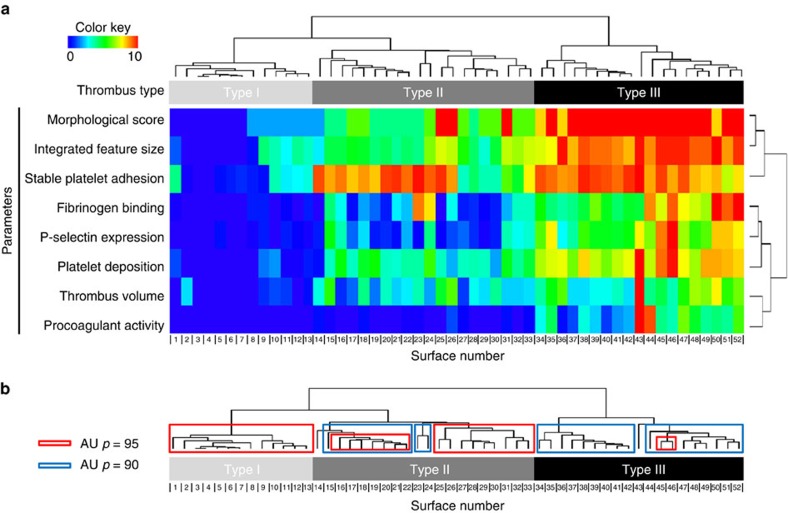
Clustering of thrombus formation at 52 microspots using eight outcome parameters. Whole blood from control subjects was perfused over arrays of microspots in a parallel-plate flow chamber for 3.5 min at 1,600 s^−1^. Numbering of coatings with different adhesive proteins or peptides as in Fig. 1. Recorded phase-contrast images were analysed for morphological score, integrated feature size and platelet deposition (surface area coverage). Following *in situ* DiOC_6_ labelling, fluorescence images were recorded to assess stable platelet adhesion (during blood flow) and thrombus volume (after blood flow). Thrombi were poststained to determine fibrinogen binding (FITC-labelled anti-fibrinogen mAb), P-selectin expression (FITC-anti-CD62P mAb), and procoagulant activity (AF647-annexin A5). Mean values of the parameters (*n*=5–7, thrombus size: *n*=4–6) were normalized from 0–10, and arranged by unsupervised hierarchical cluster analysis. (**a**) Clustered heatmap for 52 different surfaces (columns) and eight measurement parameters (rows). Clustering of surfaces revealed three different types of thrombus formation, (**b**) Robustness of data set, assessed by bootstrapping randomizations of all data with Pvclust. Shown are the *pro forma* clusters obtained, using approximately unbiased (AU) *P*-values of 90 and 95, indicative for a strong fit.

Replicate measurements of blood perfusion experiments over all 52 coated microspots and using different fluorescent labels (*n≥*4 donors per condition and label) resulted in detailed insight into the contribution of each surface to thrombus formation (see wall chart in Supplementary Fig. 2). Standardized analysis of microscopic (fluorescence) images provided the following parameters of thrombus formation: morphological score, integrated feature size, stable platelet adhesion, fibrinogen binding, P-selectin expression, overall platelet deposition, thrombus volume and procoagulant activity. Unsupervised hierarchical cluster analysis of all data (52 microspots, 8 parameters) revealed separation into three patterns of thrombus formation, indicated as types I–III (Fig. 4a). Surfaces producing type I thrombi consisted of single-protein coatings causing limited adhesion of few platelets. Type II thrombi mostly formed on surfaces co-coated with vWF or vWF-BP causing deposition of multiple platelets, single or in small aggregates, and showing limited activation (fibrinogen binding, P-selectin expression). Type III thrombi formed on several combined surfaces giving rise to large aggregates of platelets, high in activation markers. With the exception of collagen I (which binds vWF from plasma), type III thrombi only appeared at double- or triple-coated surfaces containing vWF, vWF-BP and/or laminin combined with rhodocytin or (GPO)_n_ peptides. Robustness of the unsupervised cluster analysis was checked by data re-sampling and rebuilding the tree by 10,000 randomizations with an approximately unbiased *P*-value of 90, indicative of a strong fit (Fig. 4b).

The eight output measurements clustered into adhesion-related parameters (morphological score, integrated feature size, stable platelet adhesion) on the one hand, and activation-related parameters on the other hand. Cohesion of the parameters was confirmed by multiple regression analysis (Table 1), indicating that all eight parameters contributed significantly to the clustering into type I–III thrombi (*P*<0.001). The strongest coefficients of determination for thrombus type were the linked parameters, morphological score and integrated feature size; the linked parameters, fibrinogen binding, P-selectin expression and platelet deposition; and procoagulant activity. Individually, two parameters, stable platelet adhesion and thrombus volume, were still significantly but less strongly determinative (*R*^*2*^=0.71−0.68). Complete linkage analysis after removal of the latter two parameters resulted in a cluster plot showing the same division into type I–III thrombi (Supplementary Fig. 3A,B). Overall, this analysis indicates that the distinction into three types of thrombi is not dependent on the specific measurement parameters. However, a further division into subtypes would be parameter dependent.

### Contributions of vWF and shear rate to thrombus type

**Figure 5:**
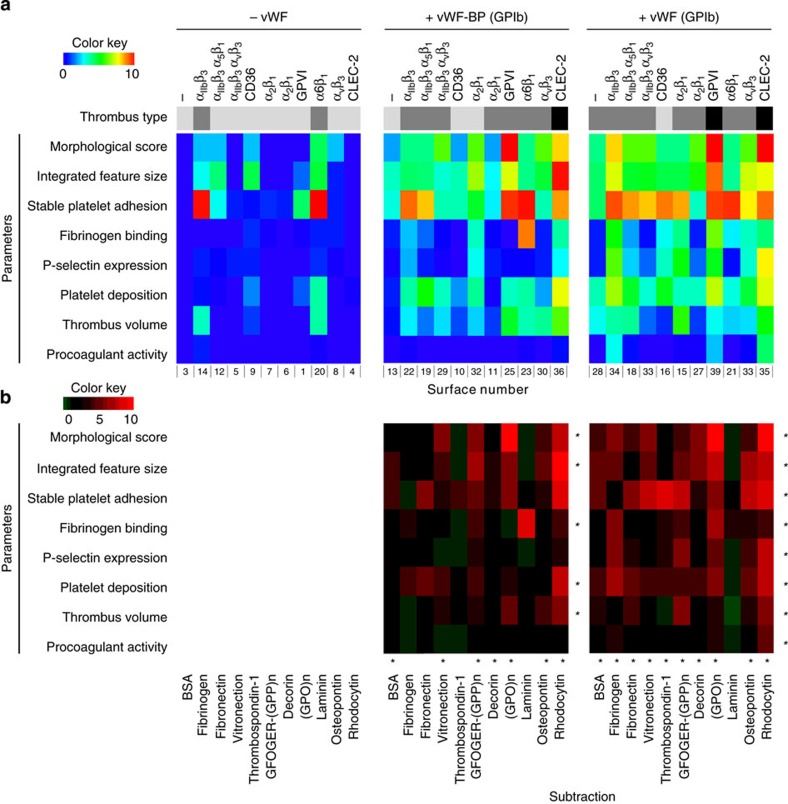
Stimulating effect of co-coating of microspots with vWF-BP or vWF. Whole blood from control subjects was perfused over arrays of microspots for 3.5 min at 1.600 s^−1^, and analysed for thrombus formation as in Fig. 1. (**a**) Sub-heatmaps of thrombus formation parameters of single coatings (left panel), co-coatings with vWF-BP (middle panel) or co-coatings with vWF (right panel). Formation of type I, II and III thrombi is represented by colour bars from grey to black. (**b**) Subtraction heatmaps, indicating the effects of co-coating with vWF-BP (middle) or with vWF (right). Colour code is from −1 to 10. *P<0.05 (two-tailed Student’s *t*-test) compared with single coating, per row or column.

Given the established role of vWF/GPIb in platelet adhesion at high-shear blood flow^2^,^21^, we performed a sub-analysis of thrombi formed on microspots co-coated with indirectly or directly GPIb-binding substances, that is, vWF-BP or vWF, respectively. The resulting heatmaps of Fig. 5a show that, for the majority of microspots, the presence of vWF-BP or vWF increased thrombus formation, as assessed from the morphological score, integrated feature size, stable platelet adhesion, platelet deposition and thrombus volume. Values of these parameters significantly increased (*P*<0.05, Student's *t*-test) compared with the surfaces without vWF (-BP), with the exception of microspots containing α_6_β_1_ ligand, laminin (Fig. 5b). Other parameters, such as fibrinogen binding and P-selectin expression, increased to a lesser extent, still significant with co-coated vWF but not vWF-BP. Procoagulant activity was not increased. Overall, the analysis identified most prominent roles of vWF (-BP) on thrombus formation co-coated with, in decreasing order, (assigned receptors in brackets): rhodocytin (CLEC-2)>(GPO)_n_ (GPVI), GFOGER-(GPP)_n_ (α_2_β_1_)>decorin, osteopontin, fibrinogen, fibronectin, vitronectin (integrins α_2_β_1_, α_5_β_1_, α_IIb_β_3_, α_v_β_3_)>laminin, thrombospondin-1 (α_6_β_1_, CD36).

**Figure 6:**
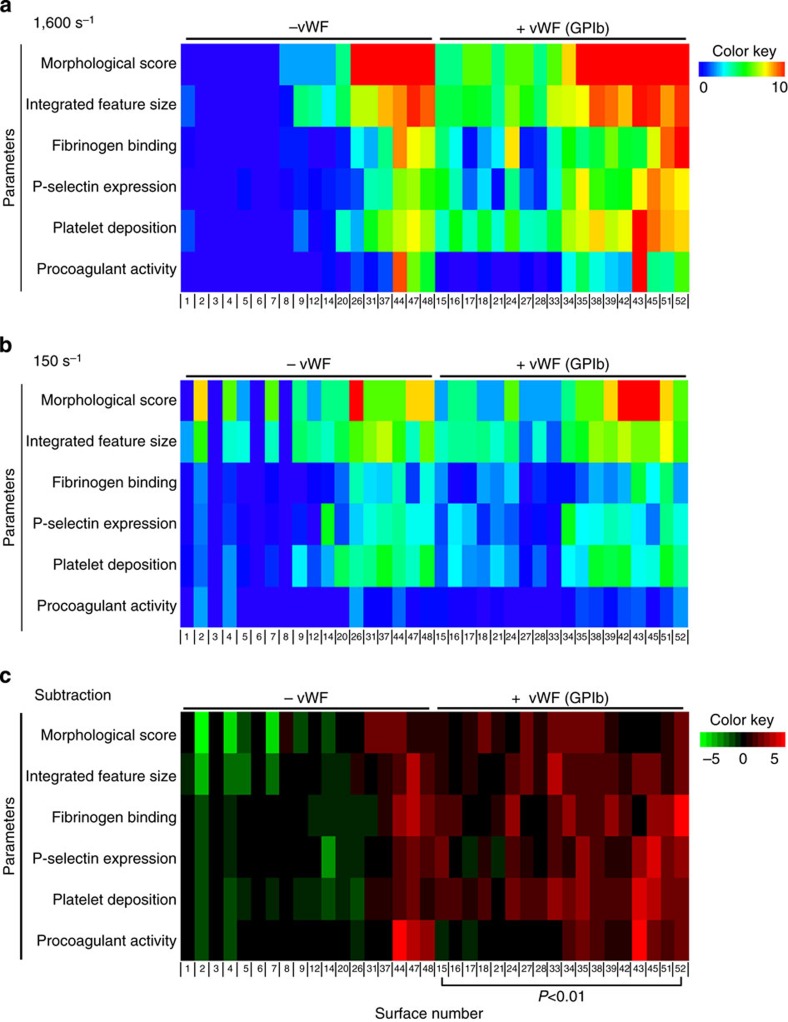
Effect of wall shear rate on thrombus formation on microspot surfaces. Whole blood was perfused over surfaces with or without vWF at indicated wall shear rates. (**a**) Measured parameters of thrombus formation at 1,600 s^−1^ (2 × 18 surfaces, clustering order as in Fig. 4). (**b**) Measurement parameters of thrombus formation at 150 s^−1^. (**c**) Linear subtraction heatmap of outcome parameters at low shear rate compared with high shear rate. *P*<0.01 (two-tailed Student’s *t*-test).

To substantiate this further, we also investigated the role of vWF by comparing thrombus formation at high (1,600 s^−1^) and low (150 s^−1^) wall shear rates, using 36 surfaces. At low shear rate, all six parameters analysed contributed to formation of type I–III thrombi with high coefficients of determination (Table 1). Heatmaps indicated that, in general, many surfaces that actively supported thrombus formation at high wall shear rate performed less well at low wall shear rate (Fig. 6a,b). On the other hand, several of the surfaces that were less active at high shear rate, particularly those not containing vWF, became more active at lower shear rate. These effects were even more apparent after subtraction analysis (Fig. 6c), pointing to improved thrombus formation at high shear rate for all vWF-containing surfaces (*P*<0.05) on the one hand. Interestingly on the other hand, combinations of laminin (α_6_β_1_), rhodocytin (CLEC-2) and GFOGER-(GPO)_n_ (GPVI, α_2_β_1_) provoked high and often increased thrombus formation in the absence of vWF at lower shear rate. Confirmation of these shear-dependent effects was obtained by perfusion studies with 19 surfaces at three wall shear rates of 150, 1,000 and 1,600 s^−1^. In general, platelet deposition at 1,000 s^−1^ was somewhat lower than at 1,600 s^−1^, the exception being laminin-containing surfaces where highest values were obtained at 1,000 s^−1^ (Supplementary Fig. 4).

### Receptor combinations in type of thrombus formation

A partial least-squares regression model was built in Matlab using all output parameters to verify the division of thrombi into three types, determined by unsupervised cluster analysis (Fig. 4). In the first model obtained, it was found that two components determined most of the variation (82.9 and 7.9%) in the data set assessing type I, II and III thrombi; furthermore, the six key parameters contributed similarly to this division. This allowed us to calculate mean values of these parameters as a proxy for the type of thrombi formed per surface and wall shear rate (Supplementary Table 2).

A second model was then built, again using partial least-squares regression data analysis, by fitting in the data obtained at low shear rate (which were not used in the first model). This model made it also possible to predict the thrombus type under low-shear conditions (Supplementary Table 2). From this analysis it appeared that 6 out of 11 surfaces, which each produced type I thrombi at high shear, formed type II thrombi at low shear. Furthermore, two out of eight vWF-containing surfaces producing type III thrombi at high shear formed type II thrombi under low-shear conditions. This class analysis confirmed the heatmap subtraction analysis of Fig. 6c.

**Figure 7:**
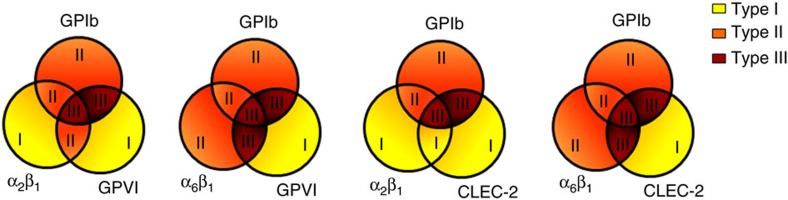
Contribution of platelet-adhesive receptors to formation of type I–III thrombi. Schematic representation of thrombus type formed at high shear rate on surfaces capturing platelets via adhesive receptors (GPIb, and integrins α_2_β_1_, α_6_β_1_) as well as signalling-linked receptors (GPVI, CLEC-2).

By combining the analysis of thrombus types and the receptor assignment matrix per surface (Fig. 1), we then evaluated the combinations of receptors contributing to formation of type III thrombi. First, computational analysis was performed for the high-shear condition of the surfaces binding GPIb (vWF, vWF-BP) in combination with the key integrins α_2_β_1_/α_6_β_1_ and the signalling receptors GPVI/CLEC-2. In combination, these three receptor/ligand classes consistently produced type III thrombi (Fig. 7). Integrins seemed to have a stimulatory but not essential role, with α_6_β_1_ being more active than α_2_β_1_. This was also clear from the mean scores of laminin-containing surfaces (α_6_β_1_ binding), which performed better than GFOGER- or decorin-containing surfaces (α_2_β_1_ binding) (Supplementary Table 2).

A similar analysis was performed by partial least-squares regression analysis linking the various receptors to thrombus type (high shear rate). For the 52 surfaces of the main heatmap, a new model was built with three components (together 72% of the variance), which after rounding gave three predicted thrombus types. A confusion matrix indicated that only 10 of the 52 samples appeared in the wrong class, with misassignment predominantly (8 out of 10) in the lower classes of thrombi (type I–II), where severity was overestimated. This model was confirmed by cross validation. Using the receptor assignments per surface, a β-weight matrix was constructed to calculate the contribution of each receptor to the type of thrombus formation (Table 2). The matrix confirmed prominent differences in contribution of the various receptors, pointing to major roles for GPIb, the signalling receptors GPVI and CLEC-2, as well as for the integrins α_6_β_1_ and α_IIb_β_3_. While contribution of α_2_β_1_ is less pronounced, the other integrins α_5_β_1_ and α_v_β_3_ along with CD36 score lowest. Note that negative values in the matrix only indicate relative inability of the receptor to contribute to type III thrombus formation. Overall, these findings identify highly stimulatory roles of CLEC-2 and α_6_β_1_ in type III thrombus formation at high shear rate, in addition to the established roles of GPIb, GPVI and α_2_β_1_.

Using the same receptor assignments and partial least-squares regression analysis, a new β-matrix was built to predict the contributions of individual receptors at low shear rate, that is, by clustering the low-shear data into the matrix of high-shear data. Cross-validation predictions showed that only 7 of the 36 surfaces were wrongly predicted for thrombus type. The resulting β-weight factors (two components, 58% of the variance) pointed to a minimized contribution of GPIb to thrombus type at low shear rate (Table 2). Further, this analysis indicated similarly high contributions of GPVI, CLEC-2, α_6_β_1_ and α_IIb_β_3_. At low shear rate, CD36 had a positive role, while α_v_β_3_ and α_5_β_1_ again did not contribute to formation of type III thrombi. Finally, the model was rebuilt by exclusion of a contribution of GPIb at low shear rate, giving essentially the same results (only 4 out of 36 surfaces wrongly predicted). This calculated absence of the GPIb-V-IX at low-shear flow conditions is in good agreement with the literature^2^.

### Platelet function disorders leading to impaired thrombus formation

A panel of nine microspot surfaces (all major receptors and vWF co-coated) was selected to determine reference values of all parameters for *n*≥6 healthy subjects (Supplementary Table 3). To assess intra-individual variability, blood samples were analysed from six healthy volunteers, taken each at four different days. For all surfaces together: intra-individual coefficient of variations (CVs) for the morphological score (5.7%) were 2.0 times lower than inter-individual CVs (11.2%); for platelet deposition, intra-individual CVs (10.8%) were 3.1 times lower than inter-individual CVs (33.7%). Similarly, for the platelet activation markers, that is, fibrinogen binding, P-selectin expression and phosphatidylserine (PS) exposure, intra-individual CVs were 3.4 times, 2.6 times and 3.5 times lower, respectively, than the corresponding inter-individual CVs.

**Figure 8:**
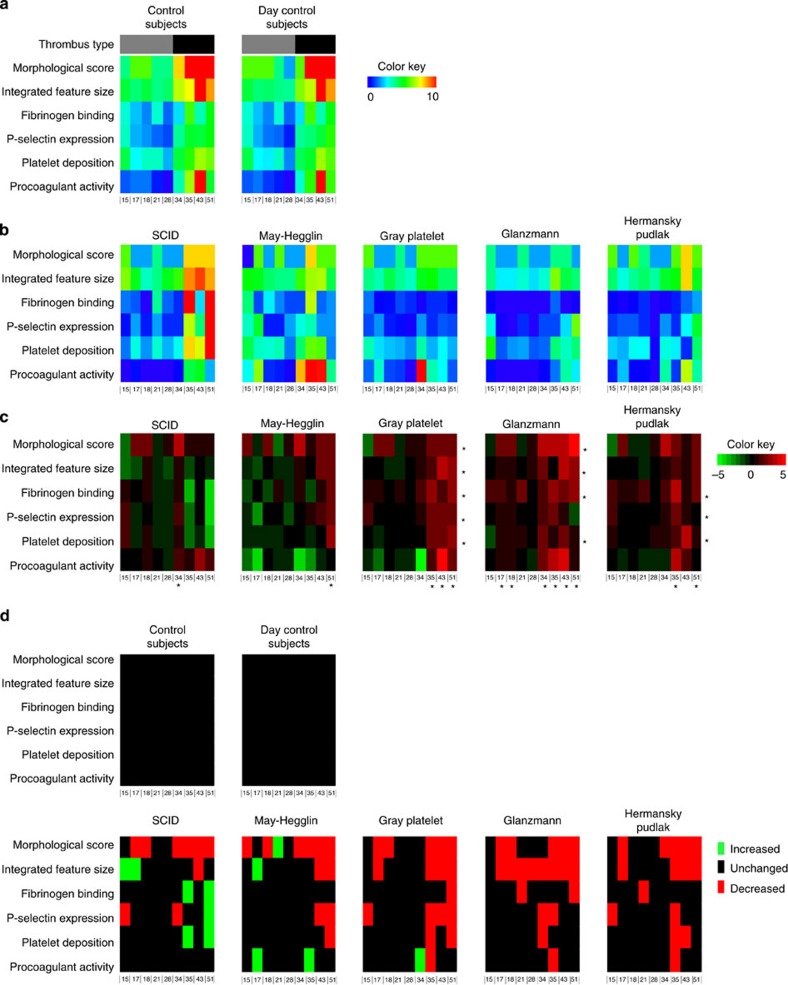
Abnormal thrombus formation in blood from patients with rare platelet function defects. Whole blood from controls subjects or indicated patients was perfused over microspots of nine different surfaces (three microspots per run, 2–3 runs per surface). Surface numbering as in Fig. 1. (**a**) Heatmap of average parameters of thrombus formation for blood samples from control subjects (left), and blood samples from day control subjects (right). (**b**) Heatmaps and (**c**) subtraction heatmaps of parameters of thrombus formation for patients with indicated syndromes: SCID, May–Hegglin anomaly, grey platelet syndrome, Glanzmann’s thrombasthenia or Hermansky–Pudlak syndrome. *P<0.05 compared with control subjects (two-tailed Student’s *t*-test). (**d**) Significance map of parameters from a representative control subject and a day control subject, as well as from all patients. Colour key: red=deviating <mean−2 s.d.; green=deviating >mean+2 s.d., relative to normal values.

Thrombus formation was also assessed in blood from rare patients with established platelet function disorders. Heatmap mean data were generated for the control subjects and the individual patients under investigation (Fig. 8a,b). These data were further evaluated in subtraction heatmaps and significance maps (Fig. 8c,d).

Thrombus formation was assessed in blood from a patient with severe immune deficiency syndrome (SCID)^40^, associated with near-complete deficiency in store-induced calcium entry in haematopoietic cells including platelets. In spite of a reduced morphological score of thrombus formation, platelet activation parameters on surfaces producing type III thrombi, that is, vWF/rhodocytin and collagen I, were increased in comparison with control subjects (Fig. 8d,e). This may point to increased CLEC-2- and GPVI-dependent platelet activation under high shear flow, for example, compensating for the absence of one of the calcium entry pathways. Blood samples were also examined from a patient with May–Hegglin anomaly^41^, characterized by a myosin cytoskeletal defect and macrothrombocytopenia, in which disease the consequences for platelet function are not well understood. Thrombus formation parameters were mostly within the normal range, with the exception of reduced platelet aggregate formation (morphological score, integrated feature size, platelet deposition) on GPVI-binding surfaces (vWF, vWF/GFOGER-(GPO)_n_, collagen I). Most values for nonGPVI-binding surfaces were in the normal range. However, platelet procoagulant activity tended to be higher on two-component surfaces.

Also investigated was blood from a patient with grey platelet syndrome^41^, phenotyped with a partial deficiency in platelet α-granules. The thrombi formed were reduced in most parameters, with the exception of procoagulant activity, typically on type III-inducing surfaces, that is, vWF/rhodocytin, vWF/GFOGER-(GPO)_n_ and collagen I (Fig. 8d). P-selectin expression was markedly reduced on all surfaces, as expected. Overall, these results may point to impaired thrombus formation due to the reduced α-granule release, in particular on surfaces activating via CLEC-2 or GPVI. Furthermore, testing the blood from a patient with Glanzmann’s thrombasthenia (deficiency in α_IIb_β_3_) resulted in a significant reduction in platelet aggregation tendency (morphological score, integrated feature size and/or fibrinogen binding) on most surfaces, thus substantiating α_IIb_β_3_ as being invariably implicated in platelet–platelet interactions. Platelet activation parameters (P-selectin expression, platelet deposition) were most prominently reduced on vWF/fibrinogen surface. A final blood sample was used from a patient with Hermansky–Pudlak syndrome^41^, characterized by absence of dense granules. In this case, parameters of thrombus formation decreased with vWF/vitronectin, vWF/rhodocytin, vWF/GFOGER-(GPO)_n_ and collagen I. Thrombus formation hence diminished on surfaces binding GPIb plus α_v_β_3_, CLEC-2 or GPVI. For all patients together, thrombus formation was mostly altered on vWF-containing surfaces with rhodocytin, GFOGER-(GPO)_n_ or collagen-I; the most affected parameters were morphological score, integrated feature size and fibrinogen binding.

### Additional role of thrombin

To determine the role of thrombin, as a potent platelet agonist in thrombus formation, recalcified blood samples were flowed over microspots containing surfaces co-coated with tissue factor. Corn trypsin inhibitor and Gly-Pro-Arg-Pro were added to prevent contact activation and fibrin polymerization, respectively. Representative results are given in Supplementary Table 4. On a vWF/fibronectin surface, all parameters of thrombus formation increased in the presence of thrombin generation. However, on coated collagen-I, platelet deposition and fluorescence markers of platelet activation parameters appeared to decrease in the presence of thrombin. This paradoxical effect was due to contraction of the thrombi on collagen, resulting in lower surface area coverage of the (labelled) platelets in the presence of thrombin. Incidental presence of fibrin clots on the surfaces made this assay variant less suitable for standardization. Overall, these data pointed to a more complex way of thrombus phenotyping in the presence of thrombin, which requires further evaluation in the future.

## Discussion

The present results aim to contribute to the recognized high need for full standardization and exploitation of flow assays for integrative whole-blood platelet function testing^9^. By systematic assessment of thrombus formation under high shear flow on 52 microspot surfaces using eight outcome parameters, we have identified the most determinative parameters of this process. These reflect overall platelet adhesion and aggregation (morphological score, integrated feature size, platelet deposition), and determinants of platelet activation (fibrinogen binding, P-selectin expression, procoagulant activity). Leaving out two parameters—stable platelet adhesion and thrombus volume—did not change the initial clustering of surfaces with different types of thrombi. The remaining six parameters contributed equally to the thrombus-forming process, and were hence combined in least-squares regression analyses to build a model for the formation of thrombi with different phenotypes. The division into three thrombus types was almost identical to that achieved by unsupervised cluster analysis of all data (52 surfaces, 8 parameters).

The cluster analysis indicated a robust distinction of surfaces into three classes, supporting: adhesion of few single platelets (type I); extensive adhesion with small aggregates and minimal platelet activation (type II); or large aggregates with fully activated platelets (type III). Thrombi of type III were only present on combined microspots containing vWF or vWF-BP, an exception being collagen I which binds vWF from plasma; other necessary components were laminin (binding α_6_β_1_), peptides containing (GPO)_n_ (binding GPVI) or rhodocytin (binding CLEC-2). Other adhesive proteins tested appeared to be less active in combined surfaces (fibrinogen>osteopontin, collagen III>fibronectin, vitronectin, thrombospondin-1, decorin). Note that the CLEC-2 ligand podoplanin is not present in the arterial wall or in plasma^35^.

A particular role in high-shear thrombus formation is played by vWF, because it binds to two abundant platelet receptors, GPIb and α_IIb_β_3_ and to several matrix proteins^2^, that is, fibrillar collagens (via its A3 domain)^19^ and, as recently demonstrated, to laminins^42^. The latter finding explains why we could not identify co-operative roles of vWF and laminin on microspots and high activity of laminin/rhodocytin co-coatings. For the majority of other surfaces, co-coating of vWF (or vWF-BP) increased the parameters of thrombus formation at high shear rate, but not at low shear rate. The platelet-activating effect of surface-immobilized laminin has been observed before under stasis, and was attributed to α_6_β_1_ and GPVI^32^,^43^. However, using GPVI inhibitors we did not find a role of GPVI in flow-dependent platelet adhesion and activation on laminin. Our data with microspotted peptides agree with the earlier conclusion that synthetic peptides designed to mimic platelet-collagen interactions via GPVI, vWF/GPIb and α_2_β_1_, namely (GPO)_n_, vWF-BP and GFOGER peptides, can completely replace native collagen fibres in supporting thrombus formation^24^,^37^,^44^.

Regression models built to predict the involvement of different platelet receptors to full-thrombus formation yielded interesting results. In addition to the established contribution of GPIb and GPVI, we find major roles for CLEC-2 and the integrins α_6_β_1_ and α_IIb_β_3_. At high shear rate, platelet adhesion via integrin α_2_β_1_ contributed little to type III thrombus formation, while other receptors α_v_β_3_, α_5_β_1_ or CD36 were even less effective. This conclusion is in full accordance with the major and additive roles of GPVI, CLEC-2 and integrin α_6_β_1_ in arterial thrombus formation in established mouse models of arterial thrombus formation *in vivo*^35^,^42^. Importantly, fitting the low-shear data into these regression models pointed to an almost annulled contribution of GPIb, which is in accordance with the current concept of the GPIb-V-IX complex as key receptor for platelet adhesion at high-shear conditions^2^. Although these results indicate a strong role of this complex in the formation of type III thrombi at high shear, they do not prove that GPIb itself triggers signalling events in platelets. According to the built model, the other most remarkable change in weight factors at low shear rate is a positive contribution of CD36.

Normal values were determined for nine surfaces and six outcome parameters. In particular, the parameters fibrinogen binding, P-selectin expression, platelet deposition and platelet procoagulant activity showed high inter-sample variation. High inter-individual variability in platelet deposition on collagen surfaces has also been reported by others, and could be correlated to plasma levels of vWF, platelet count, haematocrit, sex and platelet receptor genotype^14^. Using different types of microfluidic devices, it has also been possible to correlate outcome parameters of thrombus formation to platelet calcium responses (under stasis)^15^, and to aspirin and/or clopidogrel intake in patients with heart disease^16^. Taken together, the inter-individual variability in this kind of whole-blood measurements seems to be linked, at least in part, to clinically relevant determinants of platelet function in cardiovascular disease.

Consistent results were obtained upon application of the multi-parameter microspot assay to blood from patients with established platelet deficiencies in platelet functions. With the exception of blood from the SCID patient, the overall effect of disease on all parameters was reduced thrombus formation (9–18 out of 54 parameters reduced) (Fig. 8d). Spots of collagen I, vWF/GFOGER-(GPO)_n_ and vWF/rhodocytin were most discriminative, all normally resulting in type III thrombi. Of the type II thrombus-inducing surfaces, only vWF/vitronectin showed two reduced parameters for the majority of patients. Together, the patient data indicate that this multi-parameter, multi-surface test detects the consequences of actin cytoskeleton alterations (May–Hegglin), deficient alpha or dense granule secretion (grey platelet, Hermansky–Pudlak) and impaired α_IIb_β_3_ activity (Glanzmann). However, in the case of impaired store-operated calcium entry (SCID patient), we find a similar number of parameters decreased and increased.

In conclusion, we developed a standardized procedure to systematically test thrombus formation upon whole-blood perfusion over arrays of microspotted adhesive surface. Using systems biology approaches, we built a model predicting the roles of platelet receptors in shear-dependent thrombus formation, and generated templates to determine aberrations in this process in patients with platelet dysfunctions. This knowledge of surfaces and output parameters is pivotal in the planned design of microscope-independent flow devices. Applications of this advanced technique are numerous, not only in the profiling of patients with a (suspected) bleeding disorder or a pro-thrombotic tendency, but also in the monitoring of functional aberrations in platelet count and of antiplatelet therapy. Furthermore, in a modified way, it can be used to assess the platelet-adhesive properties under flow of blood-derived leukocytes, natural stem cells and malignant cells in cancer.

## Methods

### Materials

Sources of proteins for microspot coatings are indicated in Supplementary Table 5. VWF purified from human plasma was obtained from University Medical Center Utrecht^45^. Rhodocytin was purified by gel filtration and ion-exchange chromatography from venom of the Malayan pit viper, *Calloselasma rhodostoma*^46^. The following triple-helical peptides were synthesized as C-terminal amides and were purified by reverse-phase high-performance liquid chromatography^47^: H-GPC(GPO)_3_GFOGERGPO)_3_GPC-NH_2_ [GFOGER-(GPO)_n_]; H-GPC(GPP)_5_GFOGER(GPP)_5_GPC-NH_2_ [GFOGER-(GPP)_n_] and cross-linked collagen-related peptide, (GPO)_n_ (ref. 48); H-GPC(GPP)_5_GPRGQOGVMGFO(GPP)_5_GPC-NH_2_, collagen type III derived vWF-BP, also called VWF-III (ref. 44). BSA was obtained from Sigma; D-phenylalanyl-L-prolyl-L-arginine chloromethylketone (PPACK) from Calbiochem; DiOC_6_ from AnaSpec; corn trypsin inhibitor from Haematologic Technology; recombinant human tissue factor from Dade-Behring; Gly-Pro-Arg-Pro from Stago.

Anti-fibrinogen antibody labelled with FITC was from WAK Chemie; FITC-labelled anti-CD62P (P-selectin) mAb from Immunotech; annexin A5 labelled with Alexa fluor (AF)647 from Molecular Probes. Blocking mAbs against specific receptors came from the following sources: mAb-1950 against integrin α_2_, mAb-1976 against integrin α_v_β_3_ and mAb-1969 against α_5_β_1_ from Merck-Millipore; Fab-152 against CD36 from Santa Cruz; chimeric mAb abciximab, directed against integrins α_IIb_β_3_ and α_v_β_3_ from Centocor. Sources of single-chain Ab 10B12 against GPVI^48^, mAb 15D7 against integrin α_2_β_1_ (ref. 48) and of 6B4 Fab_2_ fragment against the vWF-binding site on GPIbα^49^.

### Microspotting of proteins and peptides

Glass coverslips (24 × 60 mm, Menzel) were degreased with 2 M HCl in 50% ethanol, and rinsed with water and saline. Using a precision mall, arrays of three consecutive microspots (3 mm centre-to-centre distance) were applied on coverslips as 0.5 μl volumes of coating proteins or peptides. Coating concentrations were optimized to give maximal static platelet adhesion, previously established in the range of 50–250 μg ml^−1^ (refs 24, 37, 45). Osteopontin, vitronectin and vWF were applied at 50 μg ml^−1^; collagens, vWF-BP, laminin and thrombospondin-1 at 100 μg ml^−1^; and other proteins and collagen-related peptides at 250 μg ml^−1^. Coated podoplanin did not support platelet adhesion, and was not used for further experiments. Sources of coated proteins and peptides are given in Supplementary Table 5. Microspots with double or triple coatings were prepared by mixing the desired proteins or peptides at the concentrations above.

Microspot-coated coverslips were incubated for 1 h in a humid atmosphere, and washed twice with saline. Coatings were verified by staining with Coomassie brilliant blue G, showing circular stained spots of ~1,000 μm in diameter for most coated proteins; only laminin and collagen I gave smaller (800 μm) and larger (1,200 μm) spots, respectively. Before flow perfusion, coverslips were blocked with HEPES buffer pH 7.45 (136 mM NaCl, 10 mM HEPES, 2.7 mM KCl, 2 mM MgCl_2_, 0.1% glucose, 1 U ml^−1^ heparin) supplemented with 1% BSA.

### Healthy control subjects and patients

Studies were approved by the local Medical Ethics Committee (Maastricht University Medical Centre, NL31480.068.10). Blood samples (10 ml) were taken from healthy control subjects and patients, who had not used antiplatelet or anti-inflammatory medication for 2 weeks. All donors gave informed consent in accordance to the Declaration of Helsinki. Healthy controls (both sexes, age 23–58 years) were not under medical care, had not experienced bleeding problems, and had normal platelet counts (200–300 × 10^9^ l^−1^) and haematocrit values (32–42%). For determination of intra-subject variability, blood samples from six healthy donors were taken on four different days, perfused and analysed.

Patients all had a well-defined platelet deficiency and mild bleeding tendency, as reported by the examining physicians: a patient with Glanzmann’s thrombasthenia with confirmed deficiency in platelet α_IIb_β_3_ expression and normal blood cell counts^50^; a patient with SCID, *ORAI1* mutation and abolished store-regulated influx in platelets^51^; a patient with May–Hegglin anomaly (*MYH9* gene mutation) displaying macrothrombocytopenia (9 × 10^9^ platelets l^−1^) and a relatively high haematocrit level (46%); a patient with confirmed Hermansky–Pudlak syndrome (*HPS3* gene mutation), characteristically lacking platelet dense granules, as confirmed by flow cytometry (280 × 10^9^ platelets l^−1^, haematocrit 31%); a patient with suspected grey platelet syndrome with major deficiency in platelet α-granules and reduced P-selectin expression, which was accompanied by macrothrombocytopenia (37 × 10^9^ platelets l^−1^, haematocrit 33%). Blood samples from every patient were run in parallel to blood samples from healthy travel controls.

### Blood collection

Blood collection was into 0.1 volume of saline containing PPACK (40 μM) and fragmin (40 U ml^−1^, final concentrations); alternatively, where indicated, collection was into 0.1 volume of sterile 129 mM trisodium citrate. Citrated blood samples were recalcified with 3.75 mM MgCl_2_ and 7.5 mM CaCl_2_ (final concentrations) in the presence of PPACK (40 μM) and fragmin (40 U ml^−1^) prior to the flow experiment. Prior to perfusion, blood samples were checked for platelet counts and the absence of visible clots.

### Flow chamber device and flow perfusion protocol

Microspot-coated coverslips were mounted onto a transparent parallel-plate flow chamber (50 μm depth, 3 mm width and 20 mm length), and pre-rinsed with HEPES buffer pH 7.45 containing 0.1% BSA. Anticoagulated whole-blood samples (400–500 μl) were perfused through the flow chamber for a time period sufficient for full-thrombus formation on collagen I spots, that is, 6 min at 150 s^−1^, 4 min at 1,000 s^−1^ and 3.5 min at 1,600 s^−1^. Where indicated, blood samples were preincubated for 5 min with DiOC_6_ (0.5 μg ml^−1^), and fluorescence images were recorded from the microspots during blood perfusion. In other cases, thrombi formed after blood flow were poststained by 2-min perfusion (1,000 s^−1^) with colour-selected combinations of the following platelet activation markers: FITC-labelled anti-fibrinogen mAb (1:100), FITC-labelled anti-P-selectin mAb (1.25 μg ml^−1^) and/or AF647-annexin A5 (0.25 μg ml^−1^), all in HEPES buffer pH 7.45 supplemented with 0.1% BSA. After 2 min of staining (stasis), unbound label was removed by a short perfusion with the same HEPES buffer. No fixative was used.

To assess the role of thrombin on thrombus formation under high-shear flow conditions, tissue factor (500 pM) was immobilized together with a dual coating of vWF with fibronectin or collagen-I. Corn trypsin inhibitor (5 μg ml^−1^) was present in the blood collecting tube to inhibit the contact activation pathway of coagulation. Blood was drawn on 0.32% trisodium citrate and Gly-Pro-Arg-Pro (5 mg ml^−1^) was added to block fibrin polymerization and thus clot formation. During the flow perfusion, the blood was recalcified with 6.3 mM CaCl_2_ and 3.2 mM MgCl_2_ to allow thrombin to be formed via co-coated tissue factor. Platelet activation markers were determined as described for the noncoagulating conditions.

### Standardized recording of microscopic images

Phase-contrast bright-field images were recorded from all three microspots during buffer perfusion immediately after outflow of the red blood cells, using an inverted Nikon Diaphot microscope, equipped with a × 40 oil-immersion objective (numerical aperture, 1.3), a × 1.8 relay lens and a CCD camera (640 × 480 pixels)^38^. For measuring platelet activation markers, the flow chamber was placed on the stage of a confocal Bio-Rad/Zeiss MRC-600 microscope, equipped with a × 60 oil-immersion objective (numerical aperture 1.4). Dual-colour confocal fluorescence 8-bit images (512 × 512 pixels) were recorded at 488 and 633 nm excitation and settings, as described previously^52^. Thrombus volume was determined using a fast line-scanning Zeiss LSM7 microscope system, equipped with a × 63 oil objective. Confocal *z*-stacks were recorded of DiOC_6_-labelled platelets in thrombi (8-bit images of 512 × 512 pixels; 106 × 106 μm, stack distance 0.5 μm). The same label and system were used to measure stable platelet adhesion during blood flow (8-bit images, taken at 2-s intervals). For each flow run, five representative microscopic images were taken from each of the microspots. Flow assays per blood sample were performed in duplicate or in triplicate, if duplicates showed marked variation.

### Quantitative analysis of recorded images

Phase-contrast and fluorescence images were analysed by standardized journals, using Metamorph software (Molecular Devices), or in the case of LSM7 images with Axiovision Rel.4.8 software (Carl Zeiss). End-stage phase-contrast images of adhered platelets were judged as follows. The morphological score was obtained by visual inspection of the platelet features per microspot: 0, no or hardly any adhered platelets; 1, multiple single-adhered platelets; 2, extensive coverage of single-adhered platelets; 3, small platelet aggregates; 4, intermediate platelet aggregates; 5, full thrombi with large-size platelet aggregates. Platelet deposition was determined as surface area coverage by using supervised image analysis journals. In brief, auto-enhanced images were filtered vertically and horizontally and thresholds were set. The resulting binary images were subjected to a close-and-open filter, which resulted in identified regions of single or clustered adhered platelets. The integrated feature size was determined as a parameter, taking into account a proportional contribution of large and small thrombi on microspots. It was defined as the cumulative contribution of squared feature areas (*f*^*2*^), ranked from small to large (1−N), to the total feature size (Σ*f*) (equation 1):


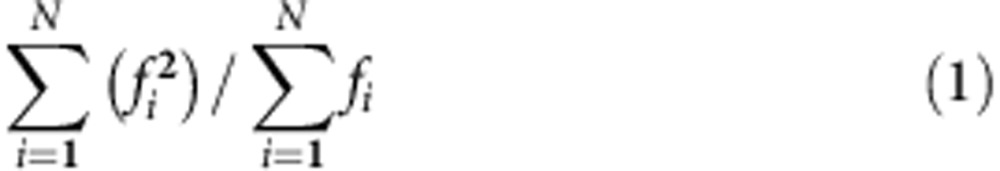


In cumulative plots, where larger size features appear at the right, the area above the curve represents a value of the integrated feature size.

Time series of images of DiOC_6_ fluorescence (1 min, 2-s intervals) were converted into subtracted, differential images using Metamorph software, and analysed for changes in pixel intensity above background^53^, thus producing a value for stable platelet adhesion. Confocal fluorescence images were thresholded with predefined journals using Metamorph software, to obtain percentage values of surface area coverage for each platelet activation label: FITC-anti-fibrinogen mAb (α_IIb_β_3_ activation), FITC-anti-P-selectin mAb (α-granule secretion; correction for non-specific labelling), AF647-annexin A5 (phosphatidylserine exposure)^38^. *Z*-stacks of confocal images of DiOC_6_ fluorescence were analysed with Axiovision software to obtain a summed thrombus volume per surface area^37^. Protocols were checked by three different observers, who were blinded to the experimental variables.

### Bioinformatics and statistics

For comparative analysis, mean values of all thrombus parameters from 52 surfaces were linearly normalized to a range from 0–10 (values of all parameters were normally distributed). Two-way unsupervised hierarchical clustering was performed using the R package version 2.3 ( www.r-project.org). Euclidean distances were calculated, and clustering was by complete linkages. Robustness of the clusters was checked using the R program, Pvclust. The clusters were rebuilt based on 10,000 randomizations, and their significance was assessed using an approximately unbiased *P*>90. Pvclust was also used to evaluate the robustness of the clusters by leaving out particular surfaces or parameters, with the aim to select those surfaces and parameters giving nonredundant information. Partial least-squares regression models with β-matrices were built in Matlab, and were employed to make 2–3-component prediction models for thrombus type and platelet receptor contribution. All models were checked by cross-validation predictions.

Parameters were correlated or compared by multiple regression analysis using the statistical package for social sciences (SPSS 19.0). Patient data were compared with normal ranges established for healthy control subjects, and statistically analysed by probability analysis. Effects of antibodies and heatmap subtractions were compared using a two-tailed Student’s *t*-test.

## Author contributions

S.M.d.W. performed experiments, analysed and interpreted data and wrote the paper; F.S. performed experiments, analysed data and revised the manuscript; M.M.E.L., R.v.K., T.M. and C.B. performed experiments and analysed data; R.C. analysed and interpreted data; A.S., I.S. and B.Z. provided essential tools; K.J., K.J.C. and R.W.F. provided essential tools and revised the manuscript; N.P. performed experiments, analysed data and revised the manuscript; J.W.M.H. and J.M.E.M.C. designed research, analysed and interpreted data and wrote the paper.

## Additional information

**How to cite this article:** de Witt, S. M. *et al.* Identification of platelet function defects by multi-parameter assessment of thrombus formation. *Nat. Commun.* 5:4257 doi: 10.1038/ncomms5257 (2014).

## Supplementary Material

Supplementary Information Supplementary Figures 1-4, Supplementary Tables 1-5 and Supplementary References

## Figures and Tables

**Table 1:** Multiple regression analysis of measurement parameters for the assessment of type I–III thrombi.

**Table 2:** Predicted contribution of platelet receptors in formation of type III thrombi at high shear rate.

**Receptor**	**Weight factor 1,600 s**^−**1**^	**150 s**^−**1**^
β constant	0.947	
GPIb-V-IX	0.687	0.049
GPVI	0.858	0.903
CLEC-2	0.763	0.964
α_6_β_1_	0.653	0.879
α_IIb_β_3_	0.527	0.877
α_2_β_1_	0.193	0.165
CD36	−0.262	0.898
α_5_β_1_	−0.102	−0.834
α_v_β_3_	−0.159	−0.746

Beta matrix values after principal component analysis of weight factors, predicting the contribution of individual adhesive receptors to formation of type III thrombi. The model built for shear rate of 1,600 s^−1^ was based on 52 surfaces and six parameters (71% of variance). A separate-scaled model was built for shear rate of 150 s^−1^, based on 36 surfaces and six parameters (58% of variance). Both models used the assignment matrix of Fig. 1. Note that negative values designate relative inactivity of the receptors to participate in type III thrombus formation.

**Figure:**