# Supplementary material for: Identification of platelet function defects by multi-parameter assessment of thrombus formation
Source: Nat Commun. 2014 Jul 16;5:4257. doi: 10.1038/ncomms5257 (PMC4109023; doi:10.1038/ncomms5257)
Supplement: Supplementary Information — Supplementary Figures 1-4, Supplementary Tables 1-5 and Supplementary References [file ncomms5257-s1.pdf]

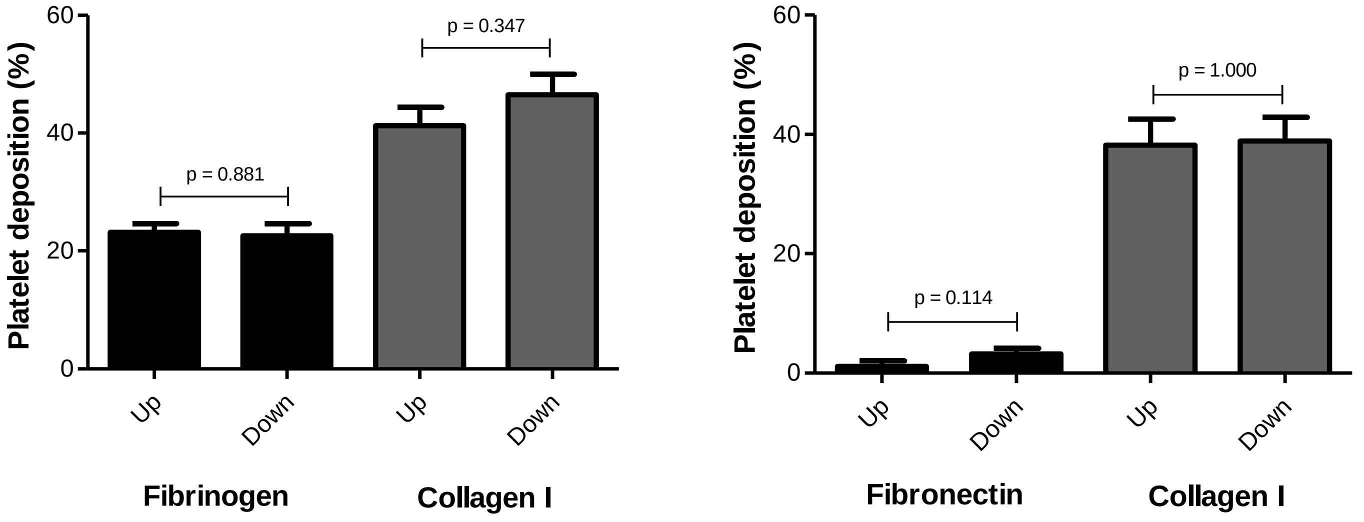

Supplementary Figure 1. Similar platelet adhesion to microspots of fibrinogen or fibronectin located upstream or downstream of collagen. Whole-blood was perfused for 3.5 min at 1600 s^{-1} over microspots containing fibrinogen, fibronectin or collagen I. Coating of microspots was at upstream or downstream locations, as indicated. Platelet deposition was analyzed as surface-area-coverage. Data are mean ± s.e.m. (n = 8; Mann-Whitney U test).

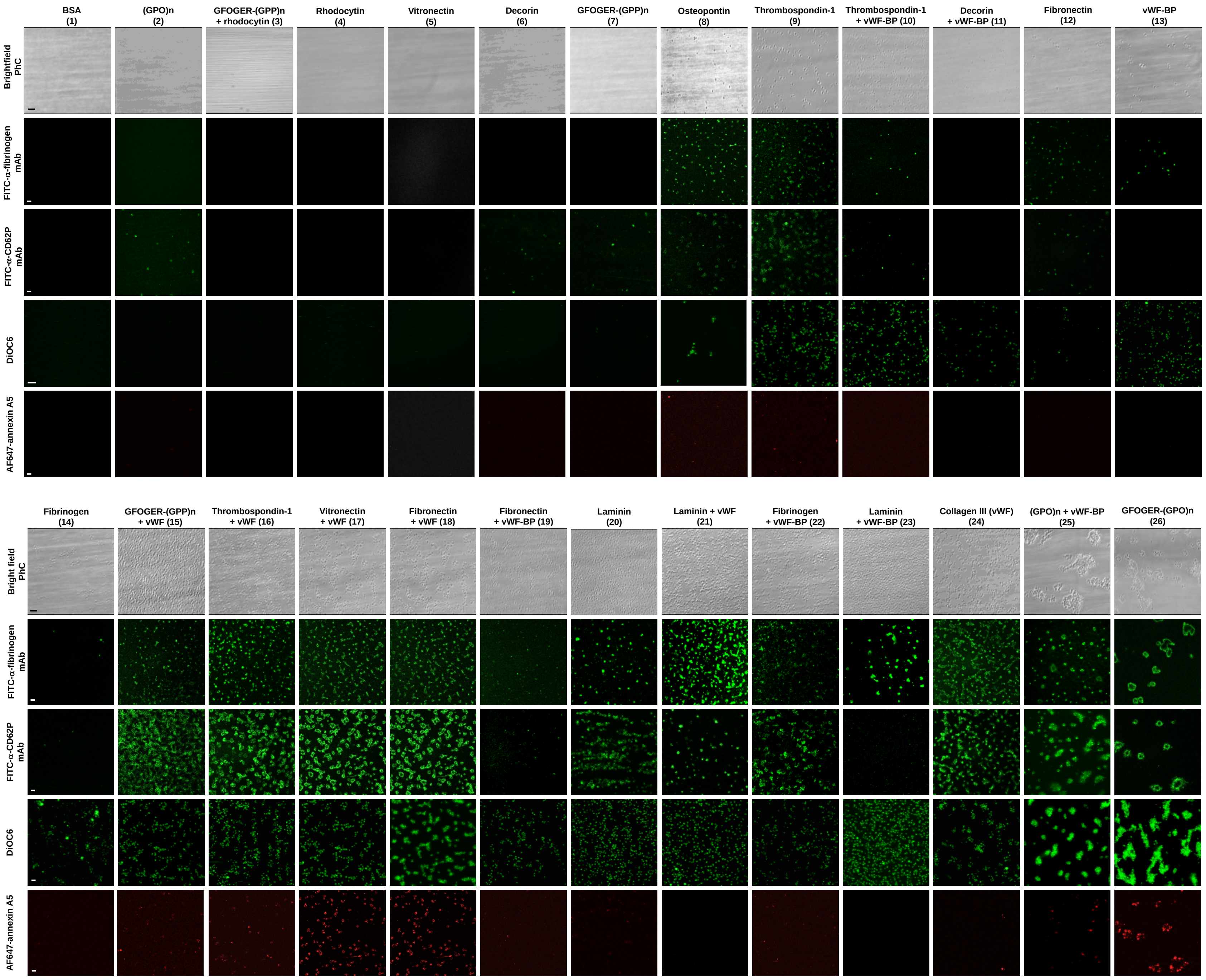
BSA (1)
(GPO)n (2)
GFOGER-(GPP)n + rhodocytin (3)
Rhodocytin (4)
Vitronectin (5)
Decorin (6)
GFOGER-(GPP)n (7)
Osteopontin (8)
Thrombospondin-1 (9)
Thrombospondin-1 + vWF-BP (10)
Decorin + vWF-BP (11)
Fibronectin (12)
vWF-BP (13)
Brightfield PhC
FITC-α-fibrinogen mAb
FITC-α-CD62P mAb
DiOC6
AF647-annexin A5
Fibrinogen (14)
GFOGER-(GPP)n + vWF (15)
Thrombospondin-1 + vWF (16)
Vitronectin + vWF (17)
Fibronectin + vWF (18)
Fibronectin + vWF-BP (19)
Laminin (20)
Laminin + vWF (21)
Fibrinogen + vWF-BP (22)
Laminin + vWF-BP (23)
Collagen III (vWF) (24)
(GPO)n + vWF-BP (25)
GFOGER-(GPO)n (26)
Bright field PhC
FITC-α-fibrinogen mAb
FITC-α-CD62P mAb
DiOC6
AF647-annexin A5

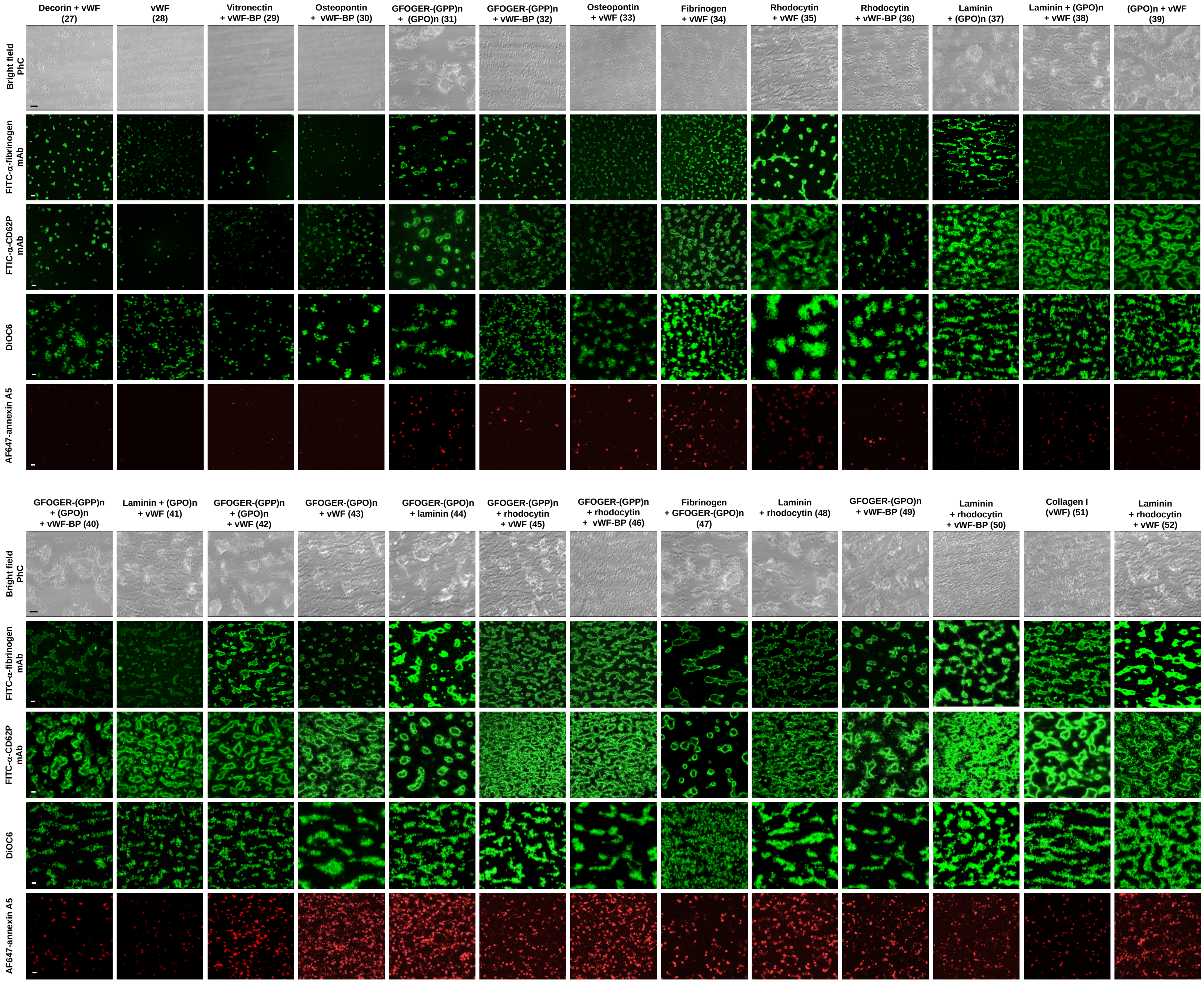
Decorin + vWF (27)
vWF (28)
Vitronectin + vWF-BP (29)
Osteopontin + vWF-BP (30)
GFOGER-(GPP)n + (GPO)n (31)
GFOGER-(GPP)n + vWF-BP (32)
Osteopontin + vWF (33)
Fibrinogen + vWF (34)
Rhodocytin + vWF (35)
Rhodocytin + vWF-BP (36)
Laminin + (GPO)n (37)
Laminin + (GPO)n + vWF (38)
(GPO)n + vWF (39)
Bright field PhC
FITC-α-fibrinogen mAb
FITC-α-CD62P mAb
DiOC6
AF647-annexin A5
GFOGER-(GPP)n + (GPO)n + vWF-BP (40)
Laminin + (GPO)n + vWF (41)
GFOGER-(GPP)n + (GPO)n + vWF (42)
GFOGER-(GPO)n + vWF (43)
GFOGER-(GPO)n + laminin (44)
GFOGER-(GPP)n + rhodocytin + vWF (45)
GFOGER-(GPP)n + rhodocytin + vWF-BP (46)
Fibrinogen + GFOGER-(GPO)n (47)
Laminin + rhodocytin (48)
GFOGER-(GPO)n + vWF-BP (49)
Laminin + rhodocytin + vWF-BP (50)
Collagen I (vWF) (51)
Laminin + rhodocytin + vWF (52)
Bright field PhC
FITC-α-fibrinogen mAb
FITC-α-CD62P mAb
DiOC6
AF647-annexin A5

Supplementary Figure 2. Wall chart of representative microscopic images of platelet adhesion and thrombus formation on 52 microspot surfaces. Whole-blood perfusion for 3.5 min at wall shear rate of 1600 s^{-1}. Numbering of surfaces is as for Fig. 1. **First rows:** phase-contrast images (115 × 115 µm) for analysis of morphological score, integrated feature size and platelet deposition (surface area coverage). **Second and third rows**: confocal fluorescence images (200 × 200 µm) captured after staining with FITC-labeled anti-fibrinogen mAb (fibrinogen binding) or FITC-labeled anti-CD62P mAb (P-selectin expression). **Fourth rows**: single plane confocal images (106 × 106 µm) of $DiOC_6$-labeled platelets, used for determination of thrombus volume. **Fifth rows**: confocal fluorescence images (200 × 200 µm) after staining with AF647-annexin A5 (procoagulant activity).

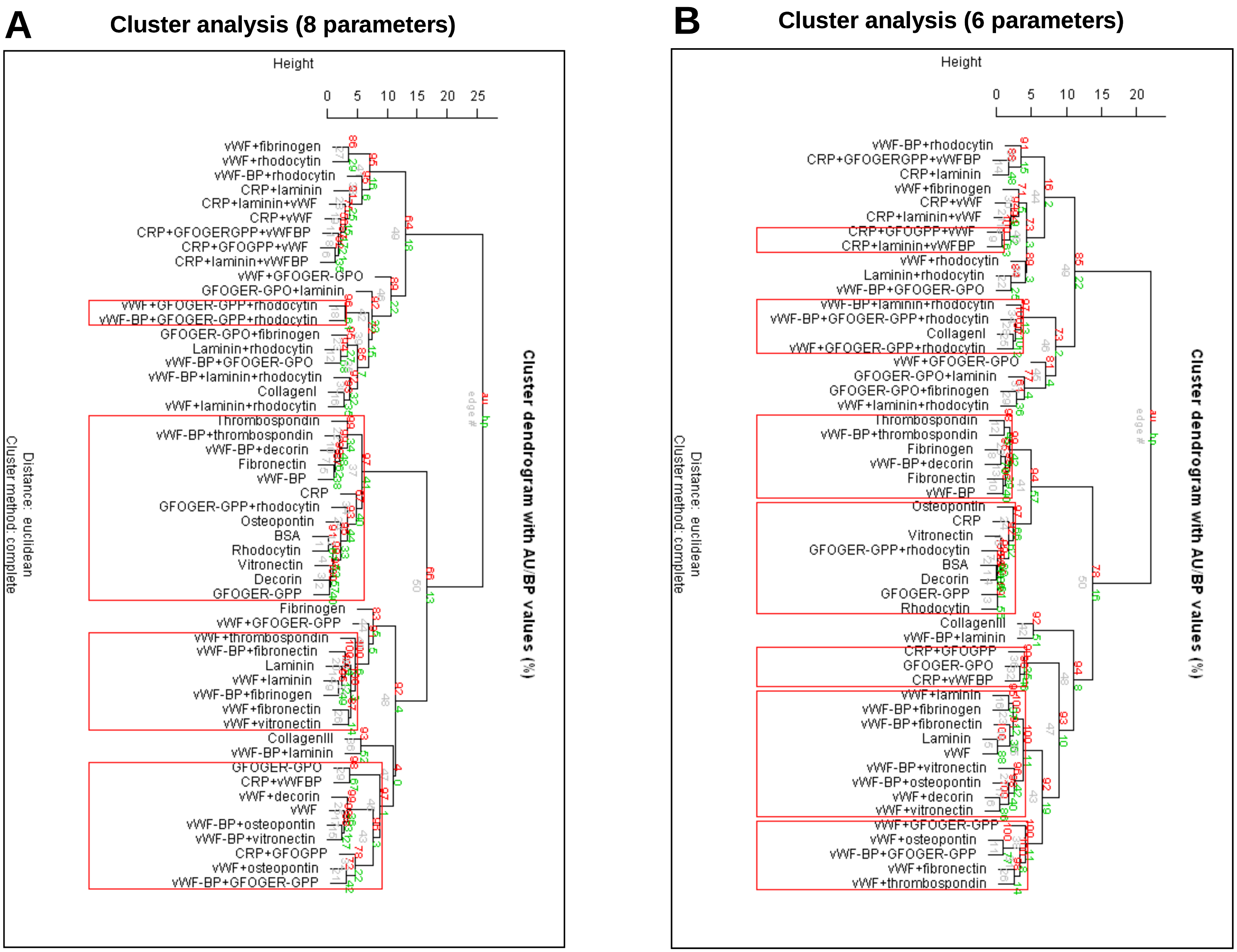

Supplementary Figure 3. Robustness of clustering of surfaces to determine thrombus type. (**A**) Unsupervised hierarchical clustering of data from 52 surfaces and 8 measurement parameters, as for Fig. 3. (**B**) Similar clustering pattern after removal of two parameters (stable platelet adhesion and thrombus volume), using complete linkage analysis by Pvclust. Note the similar division into type I-III thrombi. Red boxes indicate corresponding grouping of surfaces for either cluster analysis.

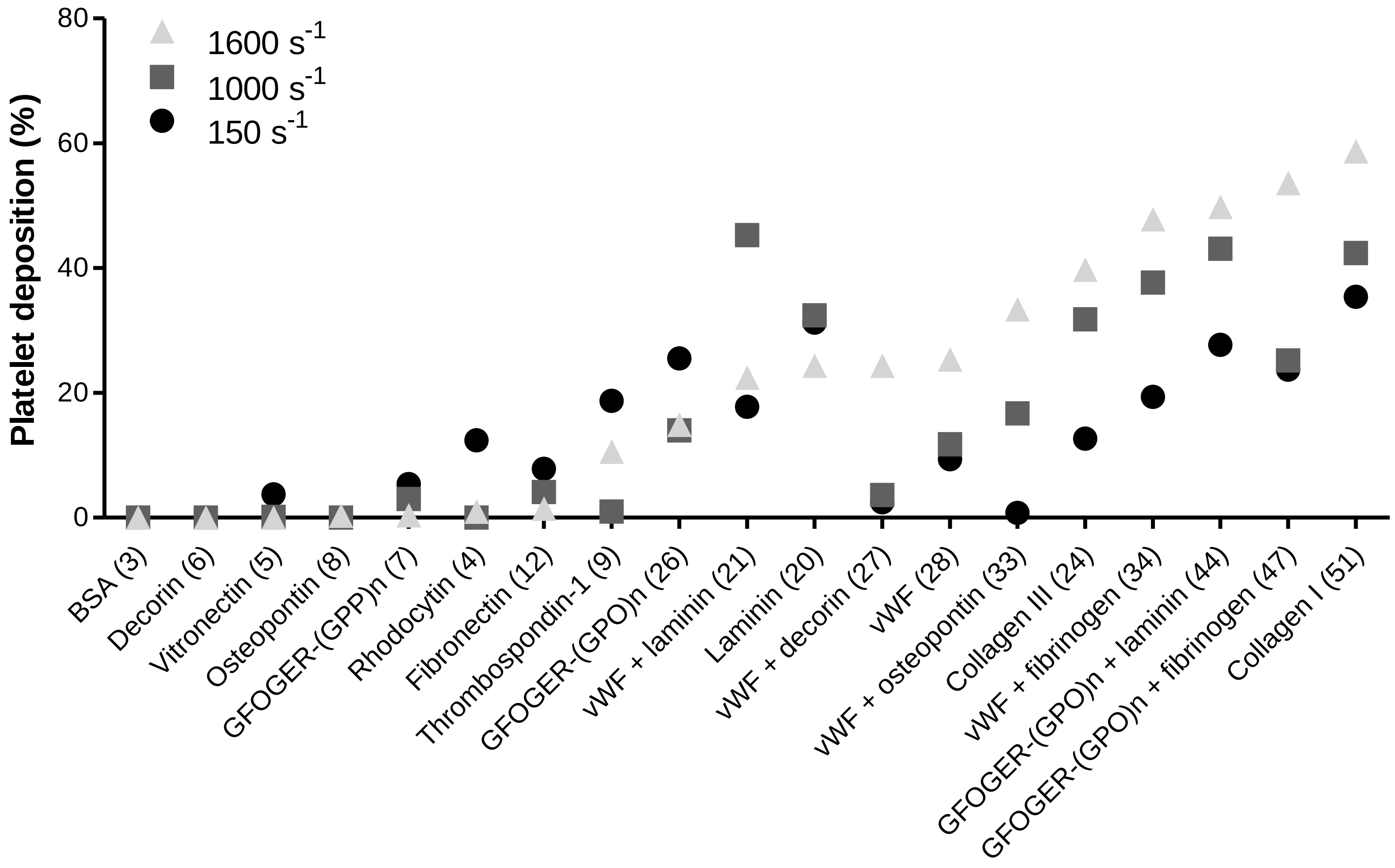

Supplementary Figure 4. Effect of increasing wall shear rate on surface area coverage by platelets. Whole blood was perfused over 19 microspot surfaces at wall shear rates of 150 s^{-1}, 1000 s^{-1} or 1600 s^{-1}. Phase-contrast images were analyzed for surface area coverage with platelets. Ranking from low to high according to change between 150 s^{-1} and 1600 s^{-1}.

Supplementary Table 1. Eexpression levels of platelet-adhesive receptors and protein/peptide ligands binding to these receptors. Only ligands used in this study are indicated. Synthetic peptide ligands are shown in italic.

Receptor	Protein/peptide ligand	Copies per platelet	Ref(s)
$\alpha_{IIb}\beta_3$	fibrinogen, fibronectin, vitronectin	80,000-100,000	[1, 2, 3, 4]
GPIb-V-IX	vWF, *vWF-BP**	30,000-40,000	[5, 6]
CD36	thrombospondin-1	10,000-25,000	[7]
$\alpha_2\beta_1$	collagen I*, III*, decorin, *GFOGER*	2,000-4,000	[8, 9, 10, 11, 12]
GPVI	collagen I, III, $(GPO)_n$	1,000-2,000	[10, 11, 12]
CLEC-2	podoplanin, rhodocytin	1,500	[13, 14, 15]
$\alpha_5\beta_1$	fibronectin	1,000	[3]
$\alpha_6\beta_1$	laminin*	1,000	[16, 17]
$\alpha_v\beta_3$	vitronectin, osteopontin	500	[18, 19]

*Binds vWF from plasma [5, 20, 21]

Supplementary Table 2. Prediction of type of thrombus formation on microspot surfaces at low shear flow conditions. Given are per surface the calculated mean parameter values for high shear rate (1600 s^{-1}) and low shear rate (150 s^{-1}). Also shown are the predictive values of mean parameters and thrombus type, obtained through model building using partial least-squares regression analysis of the high-shear data, and fitting into the model the low-shear data (UV scaled). Calculated and predicted thrombus types I, II and III are indicated in gray.

No.	Surface	**Mean parameter values (thrombus type)**			
		calculated 1600 s^{-1}	calculated 150 s^{-1}	predicted 150 s^{-1}	type difference *vs.* 1600 s^{-1}
1	$(GPO)_n$	0.46 (I)	0.44	1.09 (I)	
2	GFOGER-$(GPP)_n$ + rhodocytin	0.02 (I)	3.29	2.31 (II)	+1
3	BSA	0.10 (I)	0.00	0.84 (I)	
4	Rhodocytin	0.04 (I)	2.40	1.92 (II)	+1
5	Vitronectin	0.10 (I)	1.01	1.41 (I)	
6	Decorin	0.01 (I)	0.00	0.84 (I)	
7	GFOGER-$(GPP)_n$	0.05 (I)	1.78	1.80 (II)	+1
8	Osteopontin	0.57 (I)	0.00	0.84 (I)	
9	Thrombospondin-1	1.50 (I)	1.93	1.97 (II)	+1
12	Fibronectin	1.06 (I)	1.23	1.55 (II)	+1
14	Fibrinogen	1.07 (I)	2.52	2.05 (II)	+1
15	vWF + GFOGER-$(GPP)_n$*	3.53 (II)	1.66	1.62 (II)	
16	vWF + thrombospondin-1	3.33 (II)	2.26	2.05 (II)	
17	vWF + vitronectin*	2.71 (II)	2.08	1.91 (II)	
18	vWF + fibronectin*	3.43 (II)	1.71	1.73 (II)	
20	Laminin	2.21 (II)	2.58	2.31 (II)	
21	vWF + laminin*	2.37 (II)	1.86	1.81 (II)	
24	Collagen III (vWF)	4.96 (II)	2.59	2.13 (II)	
26	GFOGER-$(GPO)_n$	4.04 (II)	4.53	2.92 (III)	+1
27	vWF + decorin	2.85 (II)	2.81	*1.20* (I)	-1
28	vWF*	2.22 (II)	1.30	1.56 (II)	
31	$(GPO)_n$ + GFOGER-$(GPP)_n$	4.64 (II)	3.69	2.74 (III)	+1
33	vWF + osteopontin	4.13 (II)	0.70	1.17 (I)	-1
34	vWF + fibrinogen*#	6.02 (III)	2.81	2.24 (II)	-1
35	vWF + rhodocytin*	6.77 (III)	3.06	2.31 (II)	-1
37	$(GPO)_n$ + laminin	5.53 (III)	3.99	2.94 (III)	
38	vWF + $(GPO)_n$ + laminin	6.76 (III)	3.87	2.83 (III)	
39	vWF + $(GPO)_n$	6.56 (III)	4.06	2.95 (III)	
40	vWF-BP + $(GPO)_n$ + GFOGER$(GPP)_n$	5.97 (III)	3.98	3.22 (III)	
42	vWF + $(GPO)_n$ + GFOGER-$(GPP)_n$	6.26 (III)	4.70	3.22 (III)	
43	vWF + GFOGER-$(GPO)_n$*	8.66 (III)	4.60	2.96 (III)	
44	GFOGER-$(GPO)_n$ + laminin	8.30 (III)	3.99	2.69 (III)	
45	vWF + GFOGER-$(GPP)_n$ + rhodocytin	8.08 (III)	3.95	2.88 (III)	
47	GFOGER-$(GPO)_n$ + fibrinogen	7.94 (III)	3.12	2.45 (II)	-1
48	Laminin + rhodocytin	7.19 (III)	4.22	3.00 (III)	
51	Collagen I (vWF)*	7.95 (III)	4.92	3.25 (III)	
52	vWF + laminin + rhodocytin	8.49 (III)	3.68	2.59 (III)	

*Selected for patient blood analysis; #type II thrombus in unsupervised cluster analysis (Fig. 4).

Supplementary Table 3. Inter-individual variation in thrombus formation parameters for selected platelet-adhesive surfaces. Upper part: blood from 6 healthy control subjects (n = 6) was flowed over indicated surfaces at shear rate of 1600 s^{-1} (averaged from 2-3 runs). Variability is shown of parameters per surface type. Shown are raw, non-normalized data per surface (all normal distributions). **Lower part:** comparison with intra-individual coefficients of variation (CV). For intra-subject analysis, 4 blood samples from 6 healthy control subjects were flowed over the same microspot surfaces (n = 6, mean).

**Surface number**	**Morphological score**	**Platelet deposition**	**Integrated feature size**	**Fibrinogen binding**	**P-selectin expression**	**Procoagulant activity**
Inter-individual variation (raw data, mean ± s.d.)						
15	2.5 ± 0.5	37.3 ± 13.1	1.9 ± 0.2	16.2 ± 7.9	27.2 ± 8.9	1.4 ± 2.7
17	2.8 ± 0.4	23.5 ± 7.5	1.7 ± 0.1	11.6 ± 10.0	17.6 ± 13.7	1.7 ± 2.3
18	3.0 ± 0.0	28.6 ± 11.3	1.9 ± 0.3	8.6 ± 4.7	14.4 ± 10.9	1.7 ± 3.0
21	2.0 ± 0.0	28.3 ± 7.8	1.6 ± 0.2	6.3 ± 6.0	11.6 ± 9.3	0.8 ± 1.0
28	2.1 ± 0.9	17.2 ± 11.3	1.7 ± 0.3	6.6 ± 7.4	8.3 ± 6.2	0.2 ± 0.1
34	3.7 ± 0.8	39.2 ± 12.2	2.9 ± 0.5	13.2 ± 7.3	30.8 ± 10.7	4.1 ± 4.1
35	5.0 ± 0.0	46.1 ± 8.2	3.1 ± 0.2	29.8 ± 17.0	39.3 ± 8.2	10.2 ± 5.7
39	5.0 ± 0.0	56.2 ± 16.9	4.1 ± 0.1	18.2 ± 13.3	38.1 ± 11.7	11.7 ± 6.7
51	5.0 ± 0.0	52.1 ± 12.5	3.5 ± 0.3	26.3 ± 10.5	41.8 ± 8.2	5.6 ± 3.3
Inter (intra)-individual CV (%)						
15	21.9 (8.3)	35.1 (16.9)	9.8 (9.9)	48.7 (30.1)	32.8 (16.4)	2.7 (1.4)
17	14.4 (0.0)	32.0 (10.7)	4.1 (10.2)	86.0 (19.6)	77.7 (21.8)	2.3 (1.1)
18	0.0 (2.6)	39.7 (11.0)	18.1 (11.8)	55.0 (27.5)	76.0 (24.9)	3.0 (0.2)
21	0.0 (9.5)	27.4 (14.7)	12.5 (9.1)	95.2 (21.3)	80.8 (16.4)	1.0 (0.4)
28	42.0 (6.7)	66.0 (8.6)	16.4 (8.1)	112.0 (23.4)	74.6 (22.0)	0.1 (0.4)
34	22.3 (7.7)	31.2 (8.5)	17.2 (4.8)	55.2 (15.4)	34.6 (14.4)	4.1 (1.2)
35	0.0 (0.0)	17.7 (6.8)	7.4 (6.2)	57.1 (12.4)	20.9 (15.9)	10.2 (1.1)
39	0.0 (0.0)	30.0 (9.6)	2.8 (6.4)	73.1 (19.3)	30.6 (20.1)	6.7 (0.7)
51	0.0 (0.0)	24.1 (10.5)	8.9 (4.4)	40.1 (14.5)	19.6 (20.3)	3.3 (0.8)
**All**	**11.2 (3.9)**	**33.7 (10.8)**	**10.8 (7.9)**	**69.2 (20.4)**	**49.7 (19.1)**	**2.8 (0.8)***

*s.d. values.

Supplementary Table 4. Effect of thrombin generation on parameters of thrombus formation. Thrombus formation was assayed by 3.5 minutes flow of blood samples at 1600 s^{-1} over indicated microspot surfaces also containing tissue factor (0.25 fmol). Perfusion was with PPACK/fragmin anticoagulated blood (- thrombin) or with recalcified citrate-anticoagulated blood, containing 5 µg ml^{-1} corn trypsin inhibitor and 5 mg ml^{-1} GPRP (+ thrombin). Data are mean ± s.d. (n = 4; Mann-Whitney U test). * contraction and increased fluorescent intensity.

**Coated protein**	**Parameter**	**- Thrombin**	**+ Thrombin**	*P-value*
vWF/fibronectin	morphological score	3.0 ± 0.0	5.0 ± 0.0	0.008
	integrated feature size	1.9 ± 0.4	4.3 ± 0.3	0.002
	fibrinogen binding	5.9 ± 2.7	11.7 ± 1.1	0.021
	P-selectin expression	14.6 ± 9.2	28.6 ± 2.7	0.027
	platelet deposition	33.9 ± 10.8	64.4 ± 3.5	0.008
	procoagulant activity	1.0 ± 1.3	4.4 ± 3.0	0.049
Collagen I	morphological score	5.0 ± 0.0	5.0 ± 0.0	1.000
	integrated feature size	3.5 ± 0.3	3.5 ± 0.6	0.519
	fibrinogen binding	27.1 ± 10.6	7.7 ± 1.4*	0.021
	P-selectin expression	48.2 ± 7.5	21.4 ± 2.2*	0.008
	platelet deposition	58.7 ± 10.3	40.8 ± 5.7*	0.042
	procoagulant activity	4.5 ± 1.7	7.6 ± 3.8	0.705

Supplementary Table 5. Origin of proteins and peptides used for coating of microspots.

**Coated protein**	**Coating concentration ($\mu g\ ml^{-1}$)**	**Purified from**	**Company (Ref.)**
Collagen I (Horm)	100	equine tendon	Nycomed (10345787)
Collagen III	100	human placenta	Sigma Aldrich (C4407)
Decorin	250	human plasma	Sigma Aldrich (D8428)
Fibrinogen	250	human plasma	Sigma Aldrich (F3879)
Fibronectin	250	human plasma	Sigma Aldrich (F2006)
GFOGER-(GPO)n	250	chemical synthesis	Ref. [12]
GFOGER-(GPP)n	250	chemical synthesis	Ref. [12]
(GPO)n (cross-linked)	250	chemical synthesis	Ref. [12]
Laminin (511/521)	100	human placenta	Sigma Aldrich (L6274)
Rhodocytin	250	venom Malayan pit viper	Ref. [15]
Thrombospondin-1	100	human platelets	Calbiochem (605225)
Osteopontin	50	recombinant (human)	R&D Systems (1433-OP-050)
Vitronectin	50	human plasma	Sigma Aldrich (V8379)
vWF	50	human plasma	Ref. [6]
vWF-BP	100	chemical synthesis	Ref. [5]

Supplementary references

1. Savage, B., Bottini, E., Ruggeri, Z. M. Interaction of integrin αIIbβ3 with multiple fibrinogen domains during platelet adhesion. *J Biol Chem* **270**, 28812-28817 (1995).
2. Savage, B., Saldivar, E., Ruggeri, Z. M. Initiation of platelet adhesion by arrest onto fibrinogen or translocation on von Willebrand factor. *Cell* **84**, 289-297 (1996).
3. Chada, D., Mather, T., Nollert, M. U. The synergy site of fibronectin is required for strong interaction with the platelet integrin αIIbβ3. *Ann Biomed Engin* **34**, 1542-1552 (2006).
4. Zaidi, T. N., McIntire, L. V., Farrell, D. H., Thiagarajan, P. Adhesion of platelets to surface-bound fibrinogen under flow. *Blood* **88**, 2967-2972 (1996).
5. Ruggeri, Z. M., Mendolicchio, G. L. Adhesion mechanisms in platelet function. *Circ Res* **100**, 1673-1685 (2007).
6. Pugh, N., Simpson, A. M., Smethurst, P. A., de Groot, P. G., Raynal, N., Farndale, R. W. Synergism between platelet collagen receptors defined using receptor-specific collagen-mimetic peptide substrata in flowing blood. *Blood* **115**, 5069-5079 (2010).
7. Nergiz-Unal, R. *et al.* Signaling role of CD36 in platelet activation and thrombus formation on immobilized thrombospondin or oxidized low-density lipoprotein. *J Thromb Haemost* **9**, 1835-1846 (2011).
8. Fiedler, L. R. *et al.* Decorin regulates endothelial cell motility on collagen I through activation of insulin-like growth factor I receptor and modulation of α2β1 integrin activity. *J Biol Chem* **283**, 17406-17415 (2008).
9. Inoue, O., Suzuki-Inoue, K., Dean, W. L., Frampton, J., Watson, S. P. Integrin α2β1 mediates outside-in regulation of platelet spreading on collagen through activation of Src kinases and PLCγ2. *J Cell Biol* **160**, 769-780 (2003).
10. Munnix, I. C. *et al.* Collagen-mimetic peptides mediate flow-dependent thrombus formation by high- or low-affinity binding of integrin α2β1 and glycoprotein VI. *J Thromb Haemost* **6**, 2132-2142 (2008).
11. Siljander, P.R. *et al.* Platelet receptor interplay regulates collagen-induced thrombus formation in flowing human blood. *Blood* **103**, 1333-1341 (2004).
12. Smethurst, P. A. *et al.* Identification of the primary collagen-binding surface on human glycoprotein VI by site-directed mutagenesis and by a blocking phage antibody. *Blood* **103**, 903-911 (2004).
13. Bergmeier, W., *et al.* Rhodocytin (aggretin) activates platelets lacking α2β1 integrin, glycoprotein VI, and the ligand-binding domain of glycoprotein Ibα. *J Biol Chem* **276**, 25121-25126 (2001).
14. Watson, A. A. *et al.* The platelet receptor CLEC-2 is active as a dimer. *Biochemistry* 48, 10988-10996 (2009).
15. Hooley, E. *et al.* The crystal structure of the platelet activator aggretin reveals a novel $(\alpha\beta)_2$ dimeric structure. *Biochemistry* **47**, 7831-7837 (2008).
16. Inoue, O. *et al.* Laminin stimulates spreading of platelets through integrin α6β1-dependent activation of GPVI. *Blood* **107**, 1405-1412 (2006).
17. Schaff, M. *et al.* Integrin α6β1 is the main receptor for vascular laminins and plays a role in platelet adhesion, activation and arterial thrombosis. *Circulation* **128**, 541-552 (2013).
18. Asch, E., & Podack, E. Vitronectin binds to activated human platelets and plays a role in platelet aggregation. *J Clin Invest* **85**, 1372-1378 (1990).

19. Reheman, A., *et al.* Vitronectin stabilizes thrombi and vessel occlusion but plays a dual role in platelet aggregation. *J Thromb Haemost* **3**, 875-883 (2005).
20. Pareti, F.I., Niiya, K., McPherson, J.M., Ruggeri, Z. M. Isolation and characterization of two domains of human von Willebrand factor that interact with fibrillar collagen types I and III. *J Biol Chem* **262**, 13835-13841 (1987).
21. Roth, G. J., Titani, K., Hoyer, L. W., Hickey, M. J. Localization of binding sites within human von Willebrand factor for monomeric type III collagen. *Biochemistry* **25**, 8357-8361 (1986).
